# Quantifying side-chain conformational variations in protein structure

**DOI:** 10.1038/srep37024

**Published:** 2016-11-15

**Authors:** Zhichao Miao, Yang Cao

**Affiliations:** 1 Architecture et Réactivité de l′ARN, Université de Strasbourg, Institut de biologie moléculaire et cellulaire du CNRS, 67000 Strasbourg, France; 2 European Molecular Biology Laboratory, European Bioinformatics Institute, Wellcome Trust Genome Campus, Hinxton, Cambridge CB10 1SD, UK; 3 Wellcome Trust Sanger Institute, Wellcome Trust Genome Campus, Hinxton, Cambridge CB10 1SA, UK; 4 Center of Growth, Metabolism and Aging, Key Laboratory of Bio-Resource and Eco-Environment of Ministry of Education, College of Life Sciences and State Key Laboratory of Biotherapy, Sichuan University, Chengdu, 610014, China

## Abstract

Protein side-chain conformation is closely related to their biological functions. The side-chain prediction is a key step in protein design, protein docking and structure optimization. However, side-chain polymorphism comprehensively exists in protein as various types and has been long overlooked by side-chain prediction. But such conformational variations have not been quantitatively studied and the correlations between these variations and residue features are vague. Here, we performed statistical analyses on large scale data sets and found that the side-chain conformational flexibility is closely related to the exposure to solvent, degree of freedom and hydrophilicity. These analyses allowed us to quantify different types of side-chain variabilities in PDB. The results underscore that protein side-chain conformation prediction is not a single-answer problem, leading us to reconsider the assessment approaches of side-chain prediction programs.

Protein side-chain conformations have been shown to be closely related to protein mutations^1^. The protein interactions with proteins, RNA/DNA or ligands are mainly mediated by side-chain contacts. During these functional steps, some critical side-chains may change their conformations to adapt to the shape and character of its interaction partner. The ‘induced-and-fit’ model^2^ gave us plenty of examples inferring the importance of side-chain conformational change. Therefore, side-chain conformational changes, or side-chain polymorphism, could be closely related to protein functions. Nevertheless, the side-chain variations have not yet been quantitatively analyzed, the understanding of side-chain conformational variation is still intuitive and not systematic. Side-chain conformation prediction, or side-chain packing, has been a well-established problem in computational biology and involved in diverse applications, such as protein folding^3^, docking^4^, design^5^, engineering^6^ and structure optimization^7^. Various new programs^8^,^9^,^10^,^11^,^12^, which do not consider conformational change, have been proposed in recent years. Laleh *et al*.^13^ tried to predict side-chain conformation with polymorphism, but covered only a simple type of polymorphism in small scale data. Quantifying the side-chain variations can directly contribute to our understanding of structural essence of side-chain conformation and improving side-chain packing programs.

A protein structure solved by X-ray crystallography or cyro-EM is normally considered as a unique 3D conformation of the molecule in the defined condition. Molecules deposited in Protein Data Bank (PDB)^14^ generally include only unique sets of coordinates to demonstrate the 3D structures, which lead to the unique answer as ‘gold standard’ in structure prediction, such as protein structure prediction (CASP)^15^ and side-chain packing^16^. Protein side-chain conformations are usually clustered into rotamers, which are rigid conformations represented by discrete side-chain dihedral angles. However, some clues have led us to consider the variation and polymorphism^17^,^18^ of the protein side-chains: (i) the temperature factor (or B factor) of an atom describes the attenuation of X-ray scattering caused by thermal motion, and thus can be taken to indicate the relative vibrational motion of the atom; (ii) the alternate location and atom occupancy columns in PDB describe the probability of possible conformational states. Although a molecule keeps its global topology in a defined environment, the side-chain conformations are not constrained and could be flexible. With more and more crystal structures being solved, we now can find structural differences of the same protein either in the same or in different crystals. Hence, side-chain conformational variations are detected through comparing these structures of the same protein.

The first step to understand side-chain conformation is to know their immanent conformational variability in unbound state. In this work, we focus on understanding the conformational variation of protein side-chains, which do not bind nucleic acid chains. First, we described four different classes of side-chain conformations observed in crystal structures. Then, we carefully curated and analyzed several datasets of protein structures to quantify: (1) the reliabilities of the atom coordinates according to electron density; (2) the alternate location defined side-chain conformation variations; (3) side-chain conformation variations in either the same or different crystal structures and (4) the influences of backbone structure deviations and sequence mutations to side-chain variations. This work is the first quantitative analysis of side-chain conformational variations based on experimentally reliable data, providing useful knowledge of side-chain flexibility to side-chain prediction and its assessment.

Till now, protein side-chain packing methods have been widely benchmarked, considering the crystal environment^19^ or residue environments^20^. According to the conclusions of this analysis, we realized that the protein side-chain conformation prediction is not a single-answer problem and an urgent need is to reconsider the problem of side-chain packing and side-chain prediction assessment. Therefore, we propose novel approaches and large scale datasets in benchmarking side-chain packing programs considering different types of side-chain conformational variations. In perspective, this work can help us (1) to understand the flexibility of protein side-chains; (2) to optimize the side-chain prediction programs and help optimizing cryo-EM structures and (3) to relate side-chain conformational changes to protein functions in later researches.

## Results

### Side-chain conformation models

**Figure 1:**
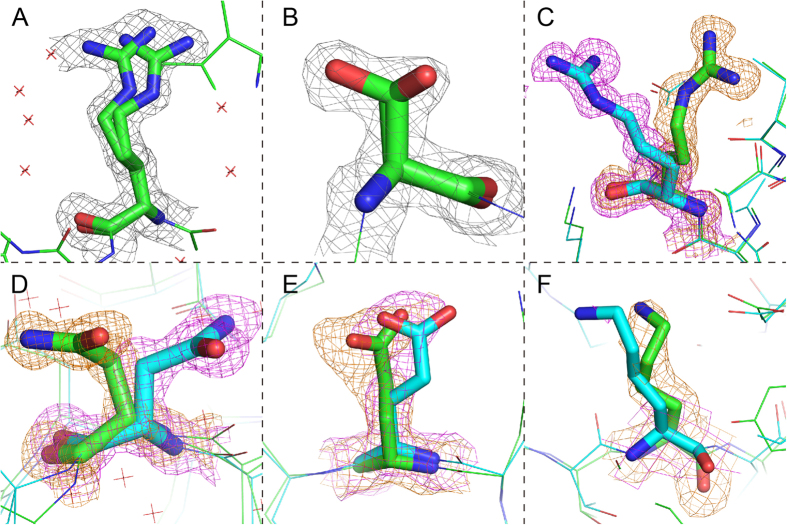
Side-chain conformational variation examples. (**A**) Arg6 in plu4264 protein (4mv2) structure has two alternate locations, resolution 1.35 Å. State A has 0.45 occupancy, while state B has 0.55 occupancy. (**B**) Ser141 in 4b1y chain B includes two discrete alternate locations of 0.66 and 0.33 occupancies. (**C**) Arg112 in lysozyme C protein (3wl2, resolution 0.96 Å) chain A (green) and B (blue). Sequence identity of the two chains is 100%, while TMscore is >0.98. Electron density in both the structures are clear, while occupancies of all the atoms equal to 1. (**D**) Asn44 in 2vb1 (green, in chain A, resolution 0.65 Å) and 1iee (cyan, in chain A, resolution 0.94 Å). Both the chains are lysozyme C protein structures in *Gallus gallus* sharing 100% sequence identity and 0.97 TMscore, solved by different labs. Electron density map of 2vb1 is shown in green and 1iee is shown in blue. Both the residues have clear electron density for all the atoms. (**E**) Glu81 in 4pj2 (green, in chain A, resolution 1.24 Å) and 3od9 (cyan, in chain A, resolution 1.41 Å). Both the chains are periplasmic lysozyme inhibitor of I-type lysozyme structures in *Aeromonas hydrophila* sharing 100% sequence identity and >0.97 TMscore, solved by the same lab. Electron density map of 4pj2 is shown in green and 3od9 is shown in blue. The residue of 4pj2 has clear electron density for all the atoms, while not all of the atoms have clear electron density in 3od9. (**F**) Lys95 in 2pa8 (green, in chain D, resolution 1.76 Å) and 2pmz (cyan, in chain (**D**), resolution 3.4 Å). Both the chains are DNA-directed RNA polymerase subunit D structures in *Sulfolobus solfataricus* sharing 100% sequence identity and >0.91 TMscore, solved by the same lab. Electron density map of 2pa8 is shown in green and 2 pmz is shown in blue. The residue of 2pa8 has clear electron density for all the atoms, while none of the side-chain atoms has clear electron density.

The coordinates of crystal structures are determined according to the electron density maps. However, some electron density maps tend to cover a larger region than a unique position. Side-chain conformations in proteins may adopt more than one conformational state, and thus, resulting ‘alternate locations’ in PDB files. We define a conformational state as a rigid set of coordinates, and a conformation as an ensemble of conformational states which can be represented by one of these states. Thus, the conformational states of a residue described by alternate locations are possible to adopt different side-chain conformations. As shown in Fig. 1A, the electron density map of the Arg side-chain covers a larger area than one single position. The PDB file defines two alternate locations of the side-chain, assigned as ‘A’ and ‘B’, where ‘A’ state has 0.45 occupancy while ‘B’ state has 0.55 occupancy. However, this is only a simplification to illustrate the conformational flexibility. According to the electron density map, any suitable conformational state between ‘A’ and ‘B’ could also be possible, yet the residue coordinates are restrained between the two conformational states.

In addition, electron density may cover discrete conformations at the same time, as illustrated in Fig. 1B. X-ray crystallography is based on the diffraction data of many molecules in a crystal, whereas the electron density map reflects all the molecules together. This implies that both conformations of the Ser are detected in this crystal.

Such side-chain alternate location is a common type of conformational variation described in single residues, but similar variations are also detected when aligning different chains of the same crystal. In Fig. 1C, two chains of the same protein in the same PDB structure have conformational variations in the Arg residue, while both of the conformational states are stable and have clear electron densities, respectively. This indicates that a residue can adopt different side-chain conformations in the same crystal environment, which is not depicted by alternate location. Further, similar variations are detected when comparing different crystal structures of the same protein. Till now, thousands of proteins have been solved with more than one crystal structures in the database showing different crystal states of the same protein. In Fig. 1D, two lysozyme C protein structures have 100% sequence identity, but the Asn44 conformations in the two structures are completely different while both of them have clear electron densities for all the side-chain atoms. If this case is similar to the alternate location case in Fig. 1B, we should also find electron densities of the other conformational state in either of the electron density map. However, even if using 0.5 sigma electron density as a cutoff, no electron density is detected in the position of the other conformer, which means both of the conformers are absolutely definite in their respective structures. This implies that even if the side-chain atoms in a PDB structure is definite (no alternate location, occupancy equals (1), the conformations could also be potentially variable.

Similar to cryo-EM, the X-ray determination of protein 3D structure has its own limitation in resolution. Thus, the electron densities of some atoms in a molecule are not clear enough. Although some side-chain conformations vary between protein structures, not all of the cases are clearly indicated in electron density map. In Fig. 1E, two structures (4pj2 in green and 3od9 in cyan) of periplasmic lysozyme inhibitor of I-type lysozyme with the same sequence and structure topology were superimposed. The Glu81 residue in 4pj2 has clear electron densities for all the side-chain atoms while the same residue in 3od9 does not. According to superimposition of the two electron density maps^21^, this residue adopts different conformations in the two PDB structures. Even if the resolution of 3od9 is as high as 1.41 Å, the conformation of this residue is not fully certain in the electron density map. Worse situations are found in structures of lower resolution. As an exemplary case in Fig. 1F, none of the side-chain atoms in the Lys95 of 2 pmz chain D (in cyan) has clear electron density. Consequently, the known experimental information is not enough to determine the side-chain conformation. When all the atom coordinates are available in a PDB file, some of them, such as Lys95 in 2pmz chain D, in fact are ‘predicted’ by crystallographers. Most likely, such side-chains are not constrained by the environment and may adopt flexible conformations.

According to our current knowledge, we conclude four types of side-chain conformations in protein structure: (1) fixed conformation, such as buried residues, that are constrained in a defined region and the coordinates are definite; (2) discrete conformation, e.g. Fig. 1B–D, different discrete conformations are all possible to be adopted; (3) cloud conformation, e.g. Arg in Fig. 1A, which may cover a limited continuous region; and 4) flexible conformation, which cannot be clearly captured by experiments, or the side-chain is intrinsically flexible in solvent. Types 2–4 are critical clues for us to appreciate side-chain conformational variations, but it is still difficult to define the boundaries between these conformational types.

### Uncertainty in PDB structure

When an electron density map of the side-chain atoms is not clear enough, information is insufficient in determining the conformation. These side-chains normally adopt the ‘flexible conformation’ and include uncertainties in crystallography. To analyze the reliability of atom coordinates and side-chain conformations, we calculated the point electron density of all the atoms. To measure the percentage of reliable atoms, atoms of electron density value >1 sigma^13^ in 2|Fo|-|Fc| map are marked as reliable, otherwise unreliable. When any of the side-chain atoms are unreliable, the side-chain conformations are also defined as unreliable (Fig. S1).

**Figure 2:**
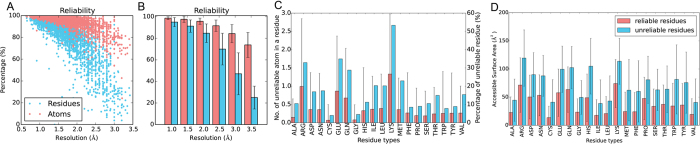
Atom reliability based on electron density, derived from dataset set1. (**A**) Percentage of reliable atoms/residues in PDB structures. X-axis is the resolution of the PDB structures, while y-axis is the percentage of the reliable atoms/residues. The percentage of reliable atoms (electron density >1sigma, only atoms from the first conformer is considered) is shown as a red dot for each protein, while the percentage of reliable residues (all atoms are reliable) is shown as a blue dot. (**B**) Average percentage of reliable atoms/residues in PDB structures. X-axis is the resolution of the PDB structures. The bars are the averaged percentages and error bars show the standard deviations. (**C**) Average percentage of reliable atoms/residues counted by residue types. Average number of atom with low electron density (sigma <1) are shown in red on left y-axis and percentages of unreliable residue (residue include more than one low electron density atom) are shown in blue right y-axis. (**D**) Histogram comparison between average residue accessible surface area of reliable and unreliable residues.

For quantitative analysis, a non-redundant set (set1, Table S1) of 3590 protein chains without nucleic acid chains bound, which are better than 3.5 Å resolution and within 25% sequence identity, were collected and analyzed. 94.8 ± 5.7% of the atoms and 81.6 ± 16.2% of the residues are reliable. Considering only side-chain atoms, the proportions drop to 90.4 ± 9.6% and 72.7 ± 15.3% (excluding Gly and Ala in residue count). This complies with the idea that backbone conformations are generally stabler than side-chains. Unsurprisingly, the percentages of reliable atom/residue decrease with the resolution value, simultaneously the standard deviations increase, as shown in Fig. 2A,B, while distributions of reliable atom/residue numbers measured by only side-chain atoms are plotted in Fig. S2. This demonstrates that low resolution structures include more uncertainties in side-chain. It is mainly because that experimental data of the lower resolution structures are not able to detect the non-fixed conformations such as alternate location. Counting the 40 structures between 3.0 and 3.5 Å, 55.5 ± 13.8% of the side-chain atoms and merely 22.1 ± 9.9% of the side-chain conformations are reliable.

**Figure 3:**
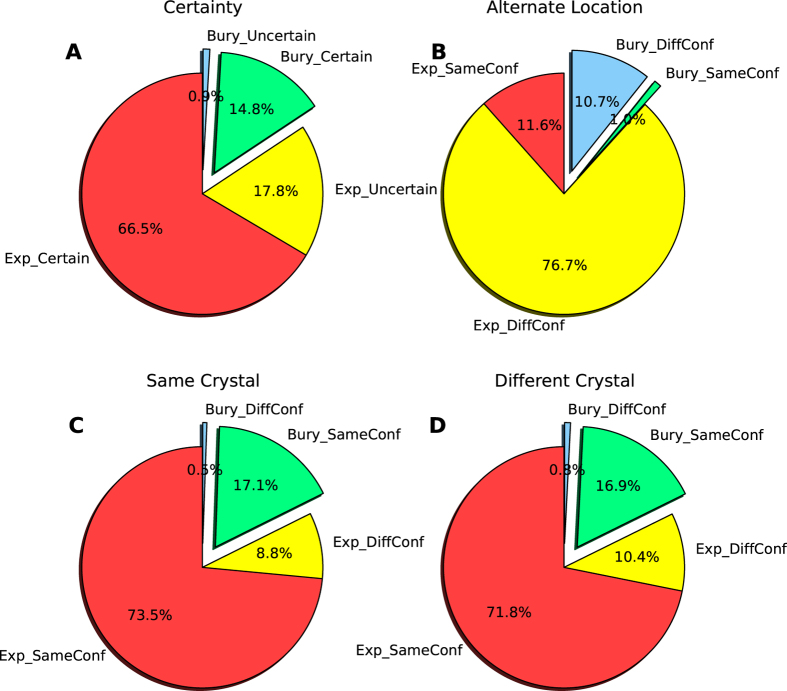
Pie plots of residue exposure and conformational variations, derived from dataset set1. (**A**) Residue exposure and certainty in electron density map. Residues with ≥1.0 Å^2^ absolute accessible surface area are defined as exposed. Exposed and certain residues are shown in red, while uncertain ones in yellow. Buried residues are plotted in the exploded pies: green show the certain ones and blue show the uncertain ones. (**B**) Residue exposure and conformational variations in alternate location residues. Side-chain conformations are defined as different when any of the χ dihedral angle changes >30°. Exposed and conformations change amongst alternate locations are shown in red, while same conformations marked in yellow. Buried and same conformations are shown in green, whereas different conformations are shown in blue. (**C**) Residue exposure and conformational variations in non-alternate location residues by comparing chains in the same crystal. Following the same coloring scheme as (**B**). (**D**) Residue exposure and conformational variations in non-alternate location residues by comparing chains in different crystals. Following the same coloring scheme as (**B**).

In Fig. 2C and Table S3, the long side-chain residue Arg, Lys, Glu, Gln and Met have more uncertain atoms and unreliable residues. At the residue level, 33% of the Arg and 53% of the Lys residues are not clear in electron density. Comparatively, aromatic residues with large side-chain groups, Phe, Trp and Tyr, are very definite in conformation. Cys has only one χ dihedral angle in side-chain and is the most reliable residue in both atom and residue level. According to the table, we can infer that the residue reliability is related to its degree of freedom (number of χ dihedral angles) rather than number of atoms in the side-chain. Residue accessibility was analyzed in Fig. 2D and observed that exposed residues include more uncertain atoms. If a buried residue is defined by its absolute accessible surface area ≥1.0 Å^2^, hardly any (<1%) buried residues are uncertain, Fig. 3A and Fig. S3. Still, more than half of the residues are exposed and clear in density map. This points out the fact that the majority of the exposed residues are generally stable in conformation excluding the cases presented in Fig. 1C,D. Interestingly, no matter how the buried residues are defined, the percentages of uncertain residues change slightly.

### Alternate locations in PDB structures

**Figure 4:**
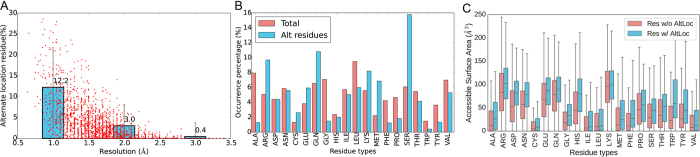
Alternate location counts, derived from dataset set1. (**A**) Percentages of alternate location residues. Each protein in the data set is presented as a red dot. X-axis is the resolution while y-axis is the percentage of residue counted in a protein. The blue histograms show the average percents of alternate location residues in proteins <1.0 Å, 1.0–2.0 Å and 2.0–3.0 Å resolution. Error bars show the standard deviations. (**B**) Residue type occurrences. Occurrence of each residue type was counted for all residues and for alternate location residues. Arg, Glu, Gln, Lys, Met and Ser show more occurrences in alternate location residues. (**C**) Average residue accessible surface area (RSA) of residues with and without alternate location atoms. Red box plots ‘Res w/o AltLoc’ show the RSA distribution of residues without alternate location atoms, while blue box plots show the RSA distribution of residues with alternate location atoms.

Alternate locations are not rare cases in crystal structures, especially in high resolution structures. In the non-redundant set, 2024 (56%) out of 3590 structures include a description of alternate location atoms. An extreme case is the PDB 4m83 of 1.7 Å resolution released in 2013 (Fig. S4), shows 20 different structure models in the same format as NMR structures, where each model is defined as an alternate location with occupancy 0.05, yet most of the residues show conformational variations. Similar cases can be found in 2ce2(1.0 Å) and 2q1z(2.4 Å). In Fig. 4A, 12.2% of the residues, on average, in protein structures within 1.0 Å resolution include alternate location atoms. But the ratio drops to 3.0% when counting structures between 1.0 and 2.0 Å, and hardly any were detected in structures >2.0 Å resolution. This implies that the electron density maps of low resolution structures (>2.0 Å) are not clear enough to determine alternate locations, while high resolution structures (<1.0 Å) can show the possible conformational variations. As resolution value increases, alternate location residue number decreases simultaneously with certainty decrease. It is probable that uncertain residues cannot be clearly defined in low resolution structures because they adopt multiple or flexible conformations.

In Fig. 4B, alternate location residues were counted by residue types and compared with overall counts. Arg, Glu, Gln, Lys, Met and Ser are more likely to include alternate locations, as the proportions of these residues obviously increase in alternate location residues. Except for Ser, all the residues include more than three side-chain dihedral angles and the conformational degree of freedom is high. This illustrates us that the alternate location is related to the conformational freedom of the residue. Ser favors two conformations at the same time as illustrated in Fig. 1B.

Alternate location is not discussed in assessing the side-chain packing programs, and normally alternate location atoms are deleted or only the atoms with alternate location indicator ‘A’ are considered^20^. However, some atoms only include indicators other than ‘A’, i.e. ‘C’ and ‘D’ two conformational states in Leu4 of 4pss chain A. Therefore, such treatment will lead to unnecessary residue missing and chain break, or omitting the possibilities of other conformational states, which affects the assessment results.

In Fig. S37, Fig. 4C and Table S10, we compared the Residue accessible Surface Area (RSA) of alternate location residues with side-chains without observable alternate locations. As a general distribution in Fig. S37, many more residues without observable alternate location are buried inside. However, the RSA of residues without alternate location covers a wider range in distribution, since many surface residues are also without observable alternate locations. According to the interquartile ranges in Fig. 4C, the RSAs of alternate location residues are larger than residues without alternate locations for all amino acid types, which implies that the exposed residues are less restrained and more likely to include alternate locations or structural variations. Still, some residues without alternate location, Arg, Lys, Glu, Gln, Asp, Asn and Pro, which are mainly hydrophilic residues, have dispersed distribution of RSAs. These residues may also exist on protein surface but without observable alternate location. This is potentially because (1) they are constrained by their interaction partners; (2) alternate location is not enough to describe all conformational variations. In the former case, the prediction of these side-chain conformations can help understanding their functional relevance; while in the latter case, side-chain flexibility should be stressed in the prediction efforts.

### Side-chain conformational variation in residues with alternate locations

**Figure 5:**
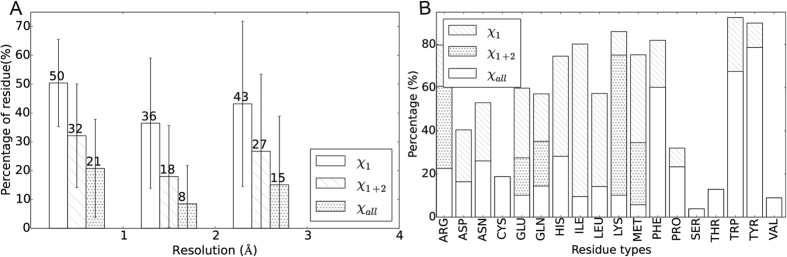
Conformational variation rate of alternate location residues, derived from dataset set1. (**A**) Percentage of alternate location residue that keep the same conformation in different conformational states counted by protein. Average values are counted for proteins <1.0 Å, 1.0–2.0 Å and 2.0–3.0 Å resolution and shown as histograms, where error bars show the standard deviations. χ_1_, χ_1+2_ and χ_all_ mean the χ dihedral angles that are kept the same in the residue. (**B**) Percentage of alternate location residue that keep the same conformation in different conformational states counted by residue types.

The side-chain conformational variations between different alternate location states were analyzed and shown in Fig. 5A. Similar to side-chain packing assessment, side-chain conformational change is defined as any of the χ dihedral angle changes more than 30°^22^. Majority of the alternate location residues adopt different conformations among different conformational states. Fewer than half of the alternate location residues keep their χ_1_ dihedral angles, while only 8% of the structures between 1.0 and 2.0 Å would completely keep their conformations among alternate locations. The percentage of residues that keep the same conformation in alternate locations slightly changes with the resolution value, while the standard deviation increases. This implies the fact that low resolution structures are less likely to keep the same conformation in alternate locations.

When a dihedral angle changes >30°, it is most likely that the residues has to adopt a different conformation rather than keep the same conformation but deviates. We can simply suppose this situation as discrete conformations, while rest of the cases as ‘cloud conformations’. Then, discrete conformations are more prevalent in alternate location residues.

Moreover, we counted the residue types of alternate location residues that change conformations in Fig. 5B. More than 60% of the aromatic residues, Phe, Trp and Tyr, tend to completely keep their conformations in different alternate locations. These aromatic residues have two χ dihedral angles and large side-chain groups. Their hydrophobicity make them more buried and less likely to change their conformations. His, Ile and Leu tend to keep the χ_1_ dihedral angle while change the other one. More than 50% Arg, Glu, Gln, Lys and Met also keep χ_1_ dihedral angle but very small part of them keep all dihedral angles.

Conformational changes in alternate location residues related to accessibility were analyzed in Fig. 3B and Fig. S5. It has been implied that alternate location residues are inclined to change their conformations in both exposed and buried residues. This complies with our knowledge of Calmodulin^23^ that buried residues are also possible to alternate their conformations.

### Side-chain conformational variations by structure comparison

#### Side-chain conformational variation in different chains of the same crystal

**Figure 6:**
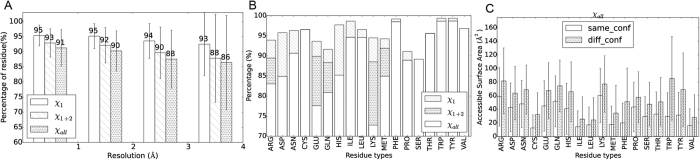
Side-chain conformations that stay the same in different chains of the same crystal, derived from dataset set2. (**A**) Percentage of residues stay the same conformation in different chains of the same crystal plotted as box plot, counted by resolutions <1.0 Å, 1.0–2.0 Å and 2.0–3.0 Å. (**B**) Percentage of residue keep the same conformation in different chains of the same crystal counted by residue types. Error bars show the standard deviations. (**C**) Average residue accessible surface area of the residues adopt same conformation (blank histograms) or different conformations (shadowed) in different chains of the same crystal.

Alternate location stands for the conformational variation in the same residue captured by crystallography. It is also interesting to know the side-chain conformational variation in different chains of the same crystal. We constructed a dataset (set2, details listed in Table S11) of 5087 non-nucleic acid binding protein pairs of different chains of identical sequences (sequence identity >90%, while 2488 pairs have the same sequences) from the same crystal structure, while TMscore^24^ are >0.8 and Cα Root Mean Square Deviation (RMSD) <3.0 Å. Alternate location residues and unreliable residues are excluded. As shown in Fig. 6A and Fig. S6, ~91% of the residues keep their side-chain conformations in different chains of the same crystal and ~95% keep the same χ_1_ dihedral angle when resolution is better than 1 Å. For the whole data set, these two values drop to 90% and 93%. The percentage of residues that keep the same conformations in different chains of the same crystal decrease with the increase of the resolution value, while the standard deviations increase. In Fig. 6B, all residue types would keep the same conformation for >70% of the counts, and all except Ser keep χ_1_ dihedral angle for >90% of the cases. ‘Long side-chain’ residues, Arg, Glu, Gln, Lys and Met, are more likely to include this type of conformational variation, followed by Asp, His, Ser and Pro. The same as the alternate location case, aromatic residues, Phe, Trp and Tyr, are not inclined to change their conformations. Need to mention, the χ_2_ dihedral angle of Pro is generally considered as correlated to χ_1_ dihedral angle and hence Pro is considered to have only one side-chain torsion angle. However, we can still find small portion of residues with the same χ_1_ dihedral angles but different χ_2_ dihedral angles.

In Fig. 6C and Figs S7 and S8, the RSAs of conformation varied residues are consistently larger than other residues that keep their conformations. This validates the concept that exposed residues are more likely to include structural variation, the same as indicated by alternate location.

#### Side-chain conformational variation in different crystals

Further, we analyzed the side-chain conformational variation of the same protein in different crystals in the same way, shown in Figs S9–S14. A dataset (set3, Table S14) of 2992 protein pairs with no binding to nucleic acid chains, from different crystals within 3.5 Å resolution but of identical sequences (sequence identity >90%, while 1868 pairs have the same sequences) and similar structural topologies (TMscore >0.8) was curated. The general distributions follow Fig. 6, drawing similar conclusions. However, fewer residues keep the same side-chain conformation in different crystals of this dataset and the percentage of the same conformation residue decreases sharper with resolution, which could be the result of conformational variations brought by crystal environment difference.

The conformational change related to residue exposure are analyzed in Fig. 3C,D and Figs S15 and S16. As alternate location residues have been excluded, we find >70% of the residues are exposed and keep their conformations in same or different crystals. Most buried residues, ~17%, adopt the same conformation in the same or different crystals. Still, ~10% of the residues tend to adopt different conformations, which part could be misleading when benchmarking side-chain prediction with single conformation.

### Side-chain conformation change related to backbone conformation change

According to the philosophy of backbone dependent rotamer library^25^, side-chain conformations also depend on their backbone torsion angles. In this analysis, the comparisons are only between proteins of similar topologies, thus, the backbone effect is minor. To quantify the influence of backbone conformation, we re-analyzed the side-chain conformations in set2 and set3 considering backbone changes. Arbitrarily, when either φ or ψ dihedral angle values of the residue backbone changes more than 30°, the backbone conformation is considered as changed. As the results of set3 shown in Fig. S17, nearly all the conformational varied residues have the same backbone conformation when the resolution is better than 2.0 Å. Despite the proportion of side-chain conformation change induced by backbone differences increase with the resolution value, the majority of the side-chain variations are not affected by the backbone effect. Fig. S18 shows a residue wise analysis drawing the same conclusion. Long side-chain residues together with Val are the least affected by backbone conformation and Cys, Ser, Asn and Asp are the most affected ones.

### Effects of sequence difference

Although >90% sequence identity point to identical proteins, residue type mutation potentially affects the conformation of its neighboring residues. This is closely related to the problem of predicting side-chain conformations with homologous structures. To evaluate the effects of sequence difference, the same analyses as Figs S9–S14 were performed on two subsets of set3 (set4 of 1868 protein pairs, Table S15, set5 of 1124 protein pairs, Table S16), which was curated by 100% sequence identity and less than 100% sequence identity, respectively. By repeating the same analyses of Figs S6–S18, Figs S19–S36 were plotted based on data sets set4 and set5. The comparisons between set4 and set5 result illustrate us the effect of sequence difference among highly homologous (sequence identity>90%) structures. According to the results shown in Figs S19–S36, very slight differences in values can be detected while the general distribution patterns are exactly the same for set4 and set5. Therefore, we conclude the effect of sequence difference on side-chain conformation is minor when sequence identity is high as 90%. It also validates the idea that backbone conformations are less likely to change than side-chain conformations. Therefore, it is also meaningful to use highly homologous structures to model protein side-chains.

## Discussion

Flexible conformations show uncertainties in structure, and thus the residue cannot be solved by X-ray crystallography resulting in low electron densities. However, such residues cover a very small portion of high resolution structures, while high resolution structures include more alternate location residues. The uncertain residues in structures with <1.0 Å resolution and in 1.0–2.0 Å are 95% and 87%, respectively. But the percentages of alternate location residue are 12% and 3%, for the corresponding <1.0 Å and 1.0–2.0 Å resolution structures. Consequently, it is estimated that 16–17% (3% + 100–87% and 12% + 100–95%) of the residues in a protein are flexible either in the form of multi-state alternate locations or flexible conformations. This number does not include the conformational difference of different chains of the same protein. Still, we can detect ≥3% of the residues show different conformations (χ_1+2_ or χ_all_) in same or different crystal structures in high resolution structures, Fig. 6A and S9. Therefore, we can roughly guess that ~20% (17% + 3%) of the protein residues may be related to conformational flexibility.

Generally, exposed residues are less restrained, but the majority of them still adopt a single conformation. Therefore, side-chain packing of these exposed residues is still a critical issue to be discussed and improved. Future efforts will lie on several problems: How exposed residues are restrained in space? Is it an artifact of crystal structure? How to predict stable conformation residues from flexible ones? Which type of residues are function related?.

Through quantitative analyses, we found side-chain conformation variation is closely related to residue type, degrees of freedom and accessibility. As shown in Table 1, long side-chain residues, Arg, Glu, Gln, Lys and Met, who include more degrees of freedom, are simultaneously hydrophilic and tend to be more exposed on protein surfaces. These residues are more apt to include conformational variation according to both the accessibility and degrees of freedom. On the contrary, aromatic residues of large hydrophobic side-chains, Phe, Trp and Tyr, are more constrained and least likely to vary their conformations. Besides, the electron density reliability is also related to side-chain conformation variations. Generally, the variable side-chains are less restrained, and thus their electron densities are less definite. The 5 types of long side-chain residues are less clear in electron density.

Side-chain conformational variation is not a minor phenomenon and can be detected in several types of descriptions or comparisons of crystal structures. Thus the benchmarking of side-chain conformation predictors is not a single-answer problem, whereas the variations can greatly affect the assessment results and conclusions. This leads us to reconsider the current assessment philosophy and metrics. Some crucial points are being overlooked: 1. Assessments without considering electron density may use some of the coordinates ‘predicted’ by crystallographers. It is necessary to remove such unreliable residues in the assessment. 2. All the alternate locations are useful, only considering the first conformational state ‘A’ is dangerous and misleading. The bias is even severer in high resolution structures, who include more alternate location atoms. Up to 12% residues could be underestimated by this treatment. 3. Assessments based on only one structure could not fully capture all the conformation probabilities and are obviously biased on the structure. A non-redundant set is normally required for benchmarking. However, removing redundancy also removes the side-chain diversity at the same time. Dataset bias is easily included when using only one ‘gold standard’. Considering these potential biases never discussed in previous benchmarks, some of the programs’ improvements in accuracies could be potentially dataset biased.

To facilitate model validation, we propose more careful benchmark approaches for side-chain packing. (1) To consider the electron density map: (A) using only reliable residues in assessment, leaving out unreliable ones; (B) superimposing the predicted structure with electron density map to measure the quality of the predicted structure model based on electron density of the coordinates of the predicted model. The later approach can cover both the cloud conformation, discrete conformations and part of the flexible/uncertain conformations. i.e. to calculate the point electron density value for each predicted atom coordinates and sum up as the assessment score. (2) To consider multiple conformations in assessment. As the cases in *RNA*-Puzzles^26^,^27^, all of the possible conformations can be used as native structures and each of them is used to compare with predicted structure, and the one nearest to the predicted structure is taken as the native structure for this predicted model. Similarly, side-chain packing quality should be assessed by the conformation closest to the predicted one. An assessment program together with all the datasets and detailed descriptions of assessment approaches are available at: https://sourceforge.net/projects/raspv180/files/Sassess/.

Currently, the side-chain packing programs reported prediction accuracies of χ_1_ and χ_1+2_ dihedral angles between 80–90%^20^. Researchers are still arguing about how to improve the prediction by one or two percent accuracy. But it seems that the limit of accuracy is related to side-chain conformational variations, while some of the best prediction programs are already close to this limit. Side-chain conformation prediction for buried residues is already in quite high accuracy, flexible residues may adopt more than just one conformation in solution and do not need special high accuracy. Exposed but single conformation side-chains are crucial in future efforts of side-chain packing. Further, side-chain prediction needs to be function related. It is much more important to predict the right conformations for the functional sites than other residues. A good prediction of side-chain conformations of functional sites can greatly benefit protein docking, protein design or virtual drug screening. The cryo-EM technique is greatly revolutionizing structural biology^28^ in recent years because of the improved resolution comparable to X-ray crystallography. However, higher resolution (<3.0 Å) X-ray structures are still better in understanding the chemical details of a molecule, especially the side-chain conformations who can be directly related to biological functions. In perspective, the side-chain packing problem will lie on how to comprehend the molecular essence of functional side-chains and to optimize side-chain conformations for exposed residues in cryo-EM structures and to relate predicted structures to function. Further, we found the sequence and backbone influences are usually minor. Hence, it could be more promising to predict side-chain conformations based on a homologous structure than completely repack all the side-chains. This can be a helpful note in protein re-design.

Finally, we may come back to the idea that proteins populate large structural ensembles and can adopt relatively flexible side-chain conformations. The central aim of side-chain packing is not to achieve a high level accuracy on datasets but to help understanding the folding essence of side-chain and protein functions mediated by side chains. Side-chain conformational variation is a very important aspect of side-chain effect and has long been overlooked. We hope to bring back its attention to help understanding protein side-chain structure.

## Material and Methods

### Data sets

Protein structures without nucleic acid chains bound, longer than 100aa and better than 3.5 Å were extracted from PDB^14^. Protein sequences were clustered with BLASTCLUST^29^. Sequence identity <25% (checked by both BLAST^30^ and PISCES web server^31^) was used as a cutoff to remove redundancy between clusters. TMscore^24^ was used to measure structure similarity.

Set1 is a non-redundant set of 3590 protein chains. Set2 is a dataset of 5087 pairs of protein chains, each pair of chains are from the same PDB structure sharing >90% sequence identity and TMscore >0.8, while sequence identity is <25% among the pairs. Set3 is a dataset of 2992 pairs of protein chains, each pair of chains are from different PDB structures (more than two letters different in PDB id). Sequence identity and TMscore cutoffs are the same as in set2. Set4 is a subset of set3, whereas each pair of chains share 100% sequence identity, while set5 is the rest of set3. Sequence identity of set3 was calculated from the sequence alignments from ClustalW2^32^. All data sets are available as supplementary information.

### Electron density

Electron density maps were downloaded from Uppsala electron density server. All the electron density maps were normalized before calculation. Point electron density was calculated by interpolation of the grid points with the Chimera^33^ library. Atoms with <1 sigma electron density are considered as unreliable. Only if all atoms in a residue are reliable, the residue is considered as reliable.

### Implementation details

Electron density map superimposition was performed by Phenix^21^. Accessible surface area is measured with NACCESS^34^ on the whole PDB structure and extracted by chain. Arg, Glu, Asp, Phe and Tyr are symmetric residues and need to be flipped in dihedral angle comparison to find the closest dihedral angle. Average B factor of side-chain atoms are average of the B factors of heavy atoms other than N, CA, C and O. Molecules in Fig. 1 are demonstrated with PyMOL^35^ and all other figures are plotted with Matplotlib^36^.

## Additional Information

**How to cite this article**: Miao, Z. and Cao, Y. Quantifying side-chain conformational variations in protein structure. *Sci. Rep*. **6**, 37024; doi: 10.1038/srep37024 (2016).

**Publisher’s note**: Springer Nature remains neutral with regard to jurisdictional claims in published maps and institutional affiliations.

## Supplementary Material

Supplementary Information

Supplementary Data S1

## Figures and Tables

**Table 1:** Features related to side-chain variations.

Residue types	Arg, Glu, Gln, Lys and Met	Phe, Trp and Tyr
Degree of freedom	High	Low
Accessibility	Exposed	Buried
Hydrophobicity	Hydrophilic	Hydrophobic
Shape	Linear	Planar
Aromatic	No	Yes
Electron density	Less clear	Clearer
