# Supplementary material for: Quantifying side-chain conformational variations in protein structure
Source: Sci Rep. 2016 Nov 15;6:37024. doi: 10.1038/srep37024 (PMC5109468; doi:10.1038/srep37024)
Supplement: Supplementary Information [file srep37024-s1.pdf]

**Supplementary Information**

# Quantifying side-chain conformational variations in protein structure

Zhichao Miao[2, 3, 4*], Yang Cao[1*]
[1]Center of Growth, Metabolism and Aging, Key Lab of Bio-Resources and Eco-Environment of Ministry of Education, College of Life Sciences, Sichuan University, Chengdu, 610064, PR China.
[2]Architecture et Réactivité de l'ARN, Université de Strasbourg, Institut de biologie moléculaire et cellulaire du CNRS, 67000 Strasbourg France
[3]European Molecular Biology Laboratory, European Bioinformatics Institute, Wellcome Trust Genome Campus, Hinxton, Cambridge CB10 1SD, UK
[4]Wellcome Trust Sanger Institute, Wellcome Trust Genome Campus, Hinxton, Cambridge CB10 1SA, UK

**Abstract**
Protein side-chain conformation is closely related to their biological functions. The side-chain prediction problem is a key step in protein design, protein docking and structure optimization. However, side-chain polymorphism comprehensively exists in protein as various types and has been long overlooked by side-chain prediction. But such conformational variations have not been quantitatively studied and the correlations between these variations and residue features are vague. Here, we performed statistical analyses on large scale data sets and found that the side-chain conformational flexibility is closely related to the exposure to solvent, degree of freedom and hydrophilicity. These analyses allowed us to quantify different types of side-chain variabilities in PDB. The results underscore that protein side-chain conformation prediction is not a single-answer problem, leading us to reconsider the assessment approaches of side-chain prediction programs.

All Supplementary information are also available on:
https://sourceforge.net/projects/raspv180/files/Sassess/Unbound/SupInfo/

**Fig S1**. Side-chain conformation with one or more atoms not clear. The Lys has a CE atom without any electron density. It is possible to adopt either of the conformation.

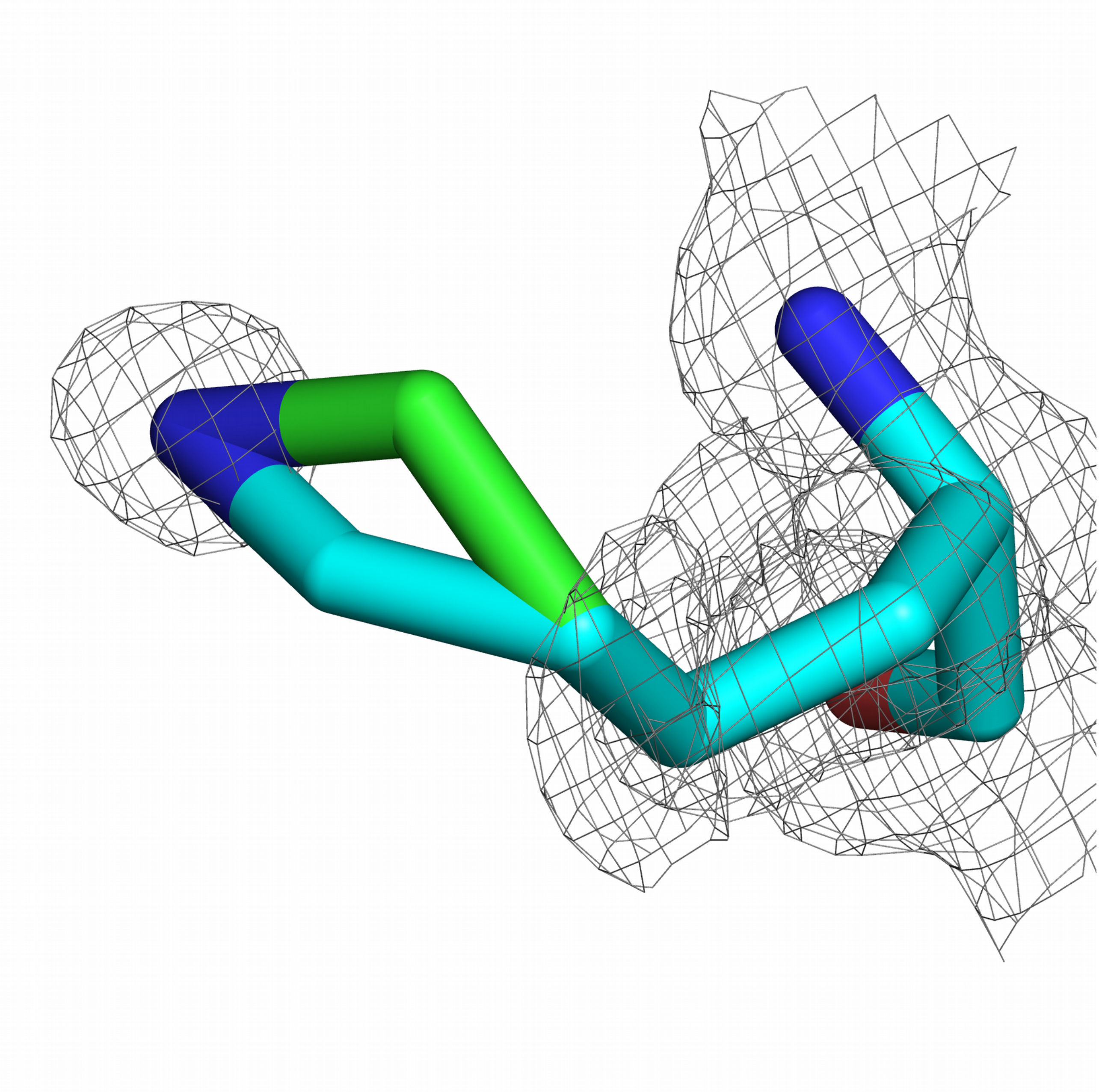

**Fig S2**. Side-chain atom reliability based on electron density (set1), counting only side-chain atoms.

A) Percentage of reliable atoms/residues in PDB structures. X-axis is the resolution of the PDB structures, while y-axis is the percentage of the reliable atoms/residues. The percentage of reliable side-chain atoms (electron density >1sigma, only atoms from the first conformer is considered) is shown as a red dot for each protein, while the percentage of reliable residues (all atoms are reliable) is shown as a blue dot.

B) Average percentage of reliable atoms/residues in PDB structures. X-axis is the resolution of the PDB structures. The bars are the averaged percentages and error bars show the standard deviations.

C) Average percentage of reliable atoms/residues counted by residue types. Average number of atom with low electron density (sigma <1) are shown in red on left y-axis and percentages of unreliable residue (residue include more than one low electron density atom) are shown in blue right y-axis.

D) Histogram comparison between average side-chain Residue accessible Surface Area of reliable and unreliable residues.

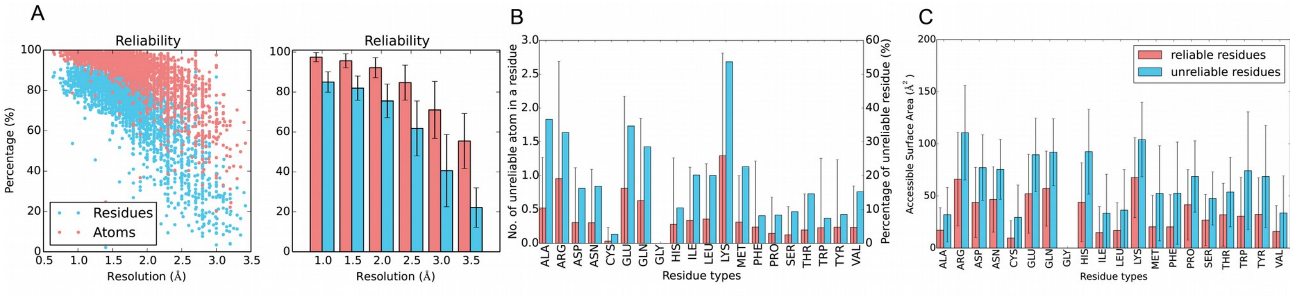

**Fig S3**. Pie plots of residue exposure and certainty (set1). Each subplot adopts a different definition of buried residue, either by all atoms or by side-chain atoms, either by absolute accessible surface area or by relative accessible surface area. 'all_abs 1.0Å$^{2}$' means buried residue is defined by absolute accessible surface area of all atoms in the residue <1.0Å$^{2}$, and 'side_rel 5%' means buried residue is defined by relative accessible surface area of side-chain atoms in the residue <5%. Red show the exposed and uncertain residues, yellow show the exposed and certain residues, green show the buried and uncertain residues and blue show the buried and certain ones.

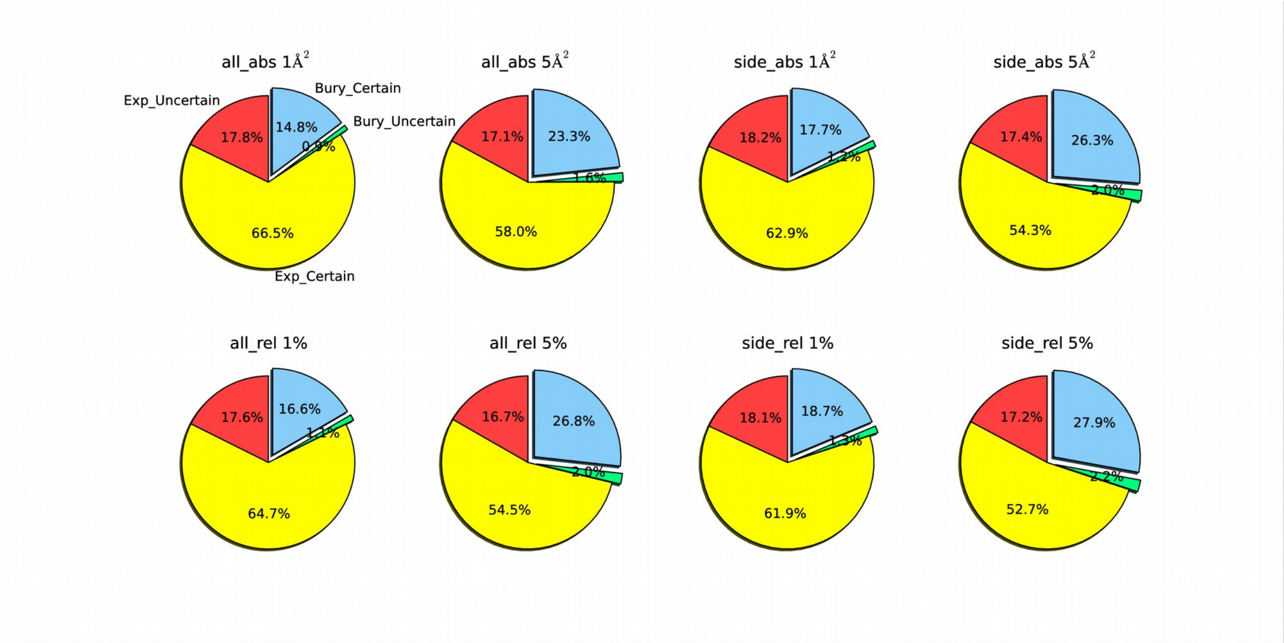

**Fig S4**. Structure of 4m83, a 20 models alternate location case.

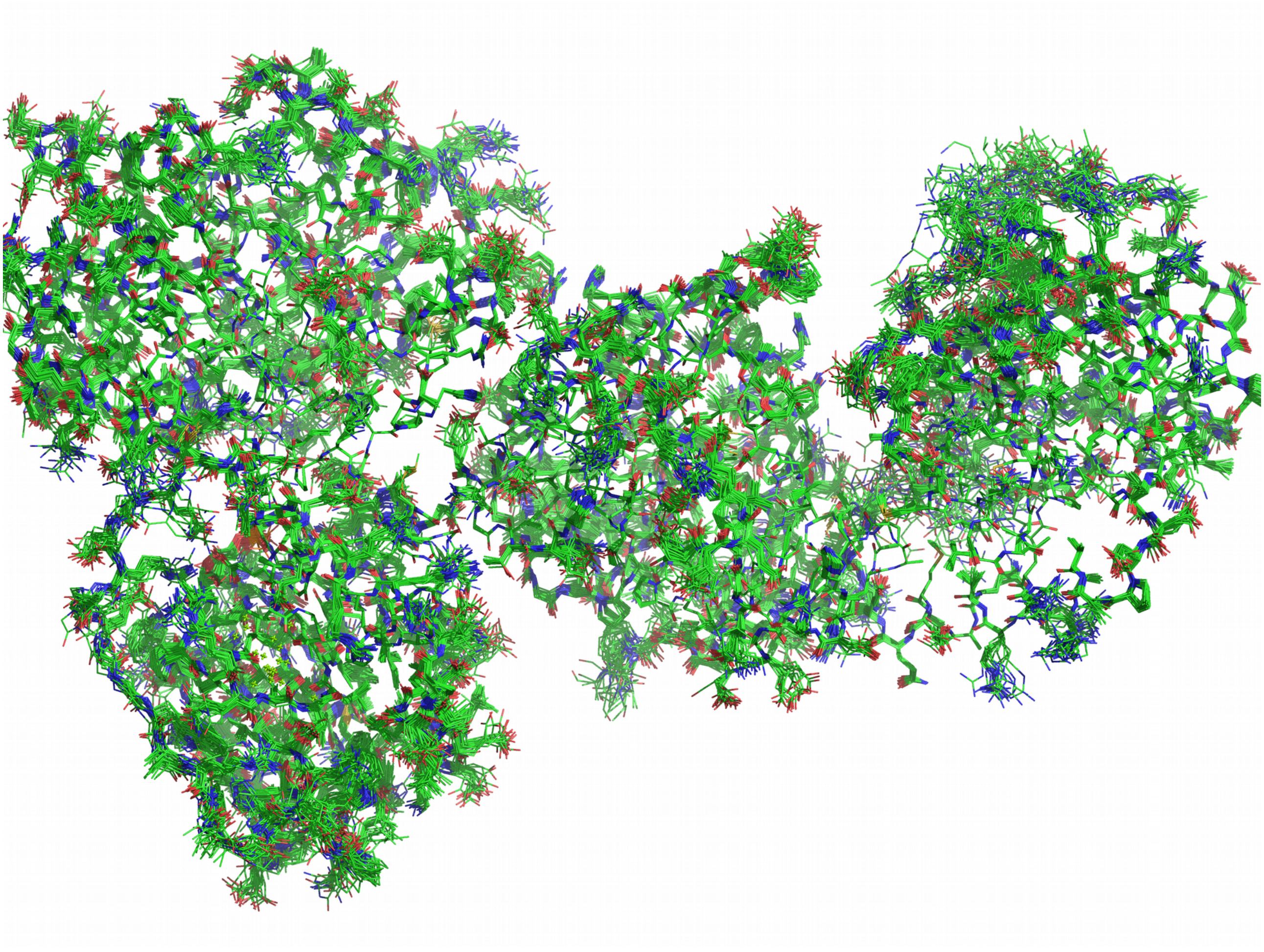

**Fig S5**. Pie plots of residue exposure and alternate location (set1).

A) Any of the χ dihedrals change is defined as conformational change. Exposed and conformations change amongst alternate location states are shown in red, while same conformations marked in yellow. Buried and same conformations are shown in green, whereas different conformations are shown in blue.

B) $\chi_1$ dihedral change is defined as conformational change, following the same coloring scheme.

C) $\chi_1$ or $\chi_2$ dihedral change is defined as conformational change.

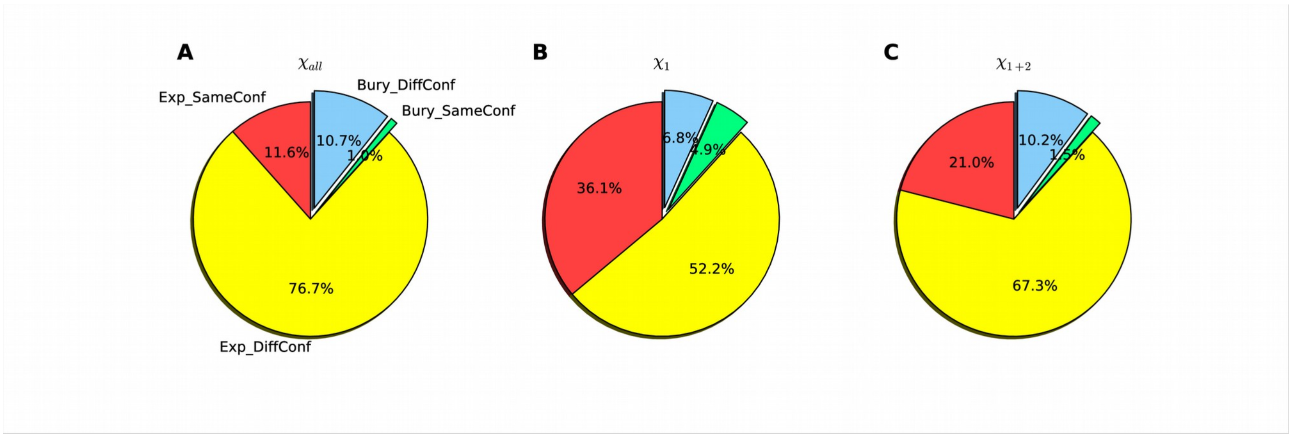

**Fig S6**. Percentage of residue that adopt same conformation in different chains of the same crystal (set2) as dot plot. Red dots show the residues that keep all the side-chain dihedral conformations, cyan dots show the residues that keep $\chi_1$ and $\chi_2$ dihedrals within 30°, while green dots are residues only keep $\chi_1$.

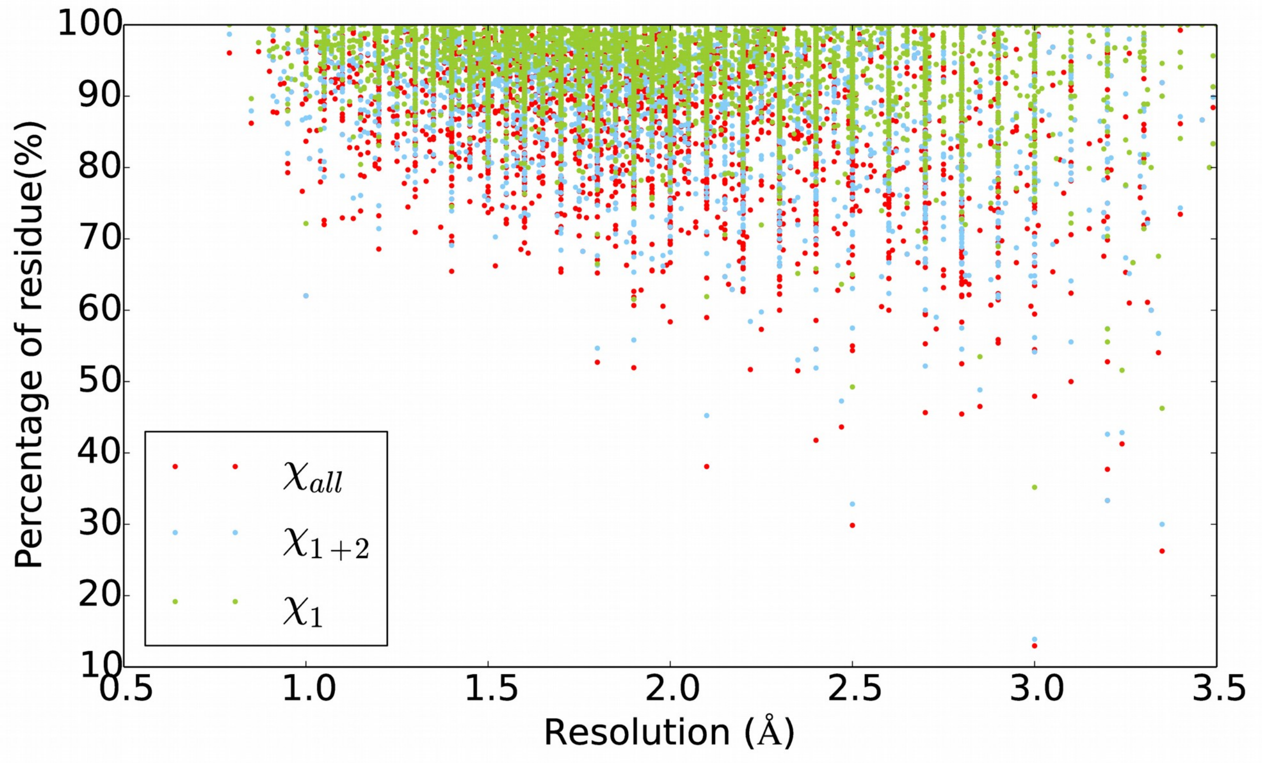

**Fig S7**. Average residue accessible surface area of the residues adopt same $\chi_1$ dihedral (blank bars) or different $\chi_1$ dihedral angles (dotted bars) in different chains of the same crystal. Error bars show standard deviations.

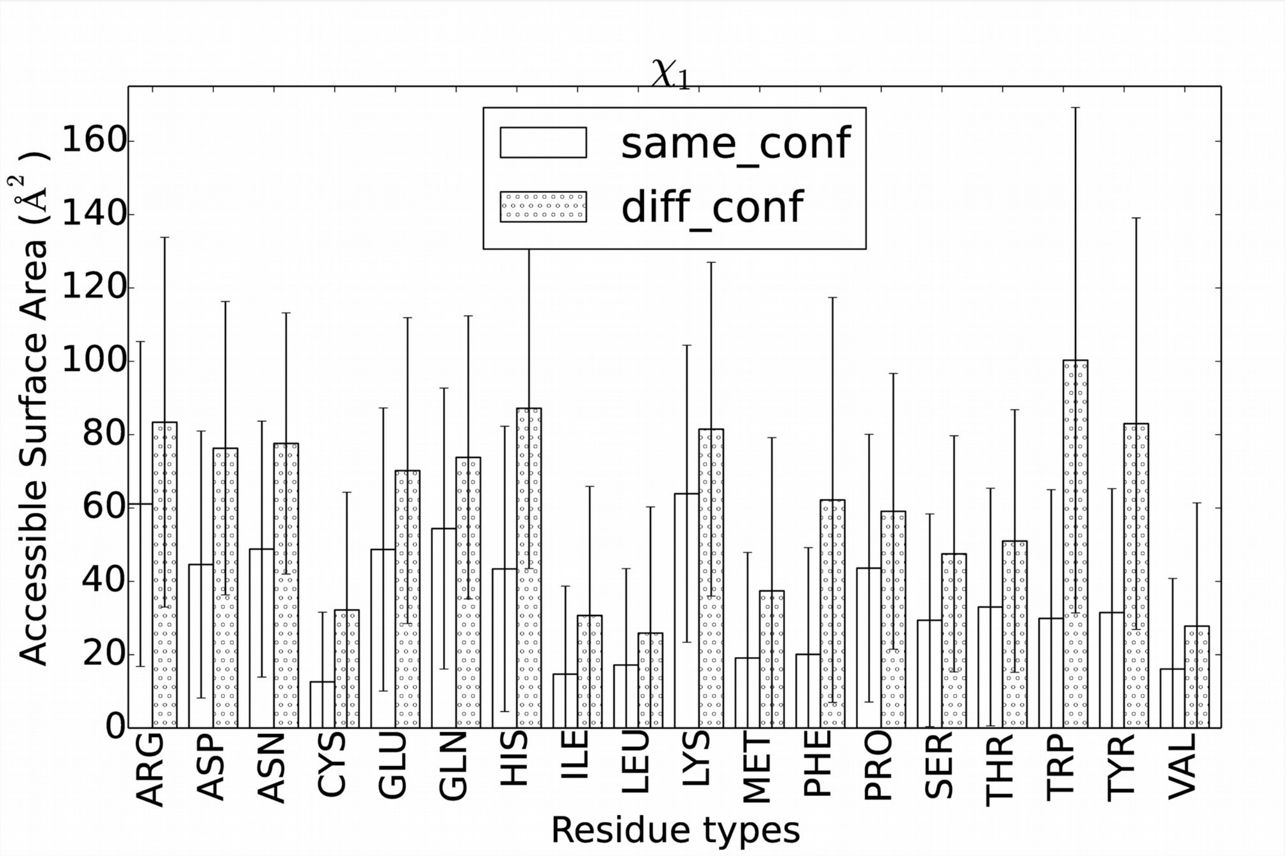

**Fig S8**. Average residue accessible surface area of the residues adopt same $\chi_1$ and $\chi_2$ dihedral (blank bars) or different $\chi_1$ and $\chi_2$ dihedral angles (dotted bars) in different chains of the same crystal (set2). Error bars show standard deviations.

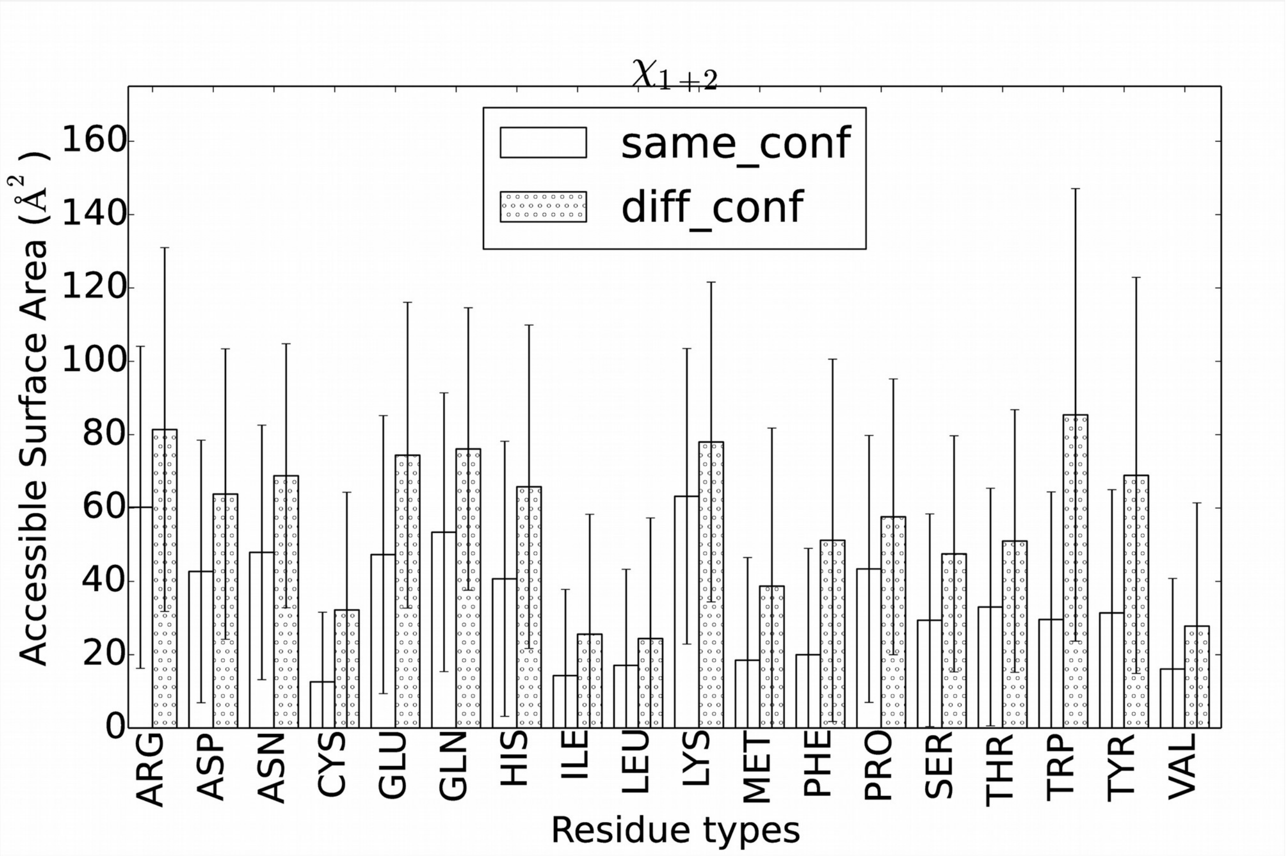

**Fig S9**. Average percentage of residue stay the same conformation in different crystals of the same protein (set3), counted by resolution <1.0Å, 1.0-2.0Å, 2.0-3.0Å and >3.0Å. Blank bars are $\chi_1$ dihedral stays the same, dashed bars are $\chi_1$ and $\chi_2$ dihedrals keep the same conformations, while dotted bars are residues keep all the side-chain conformations. Error bars show standard deviations.

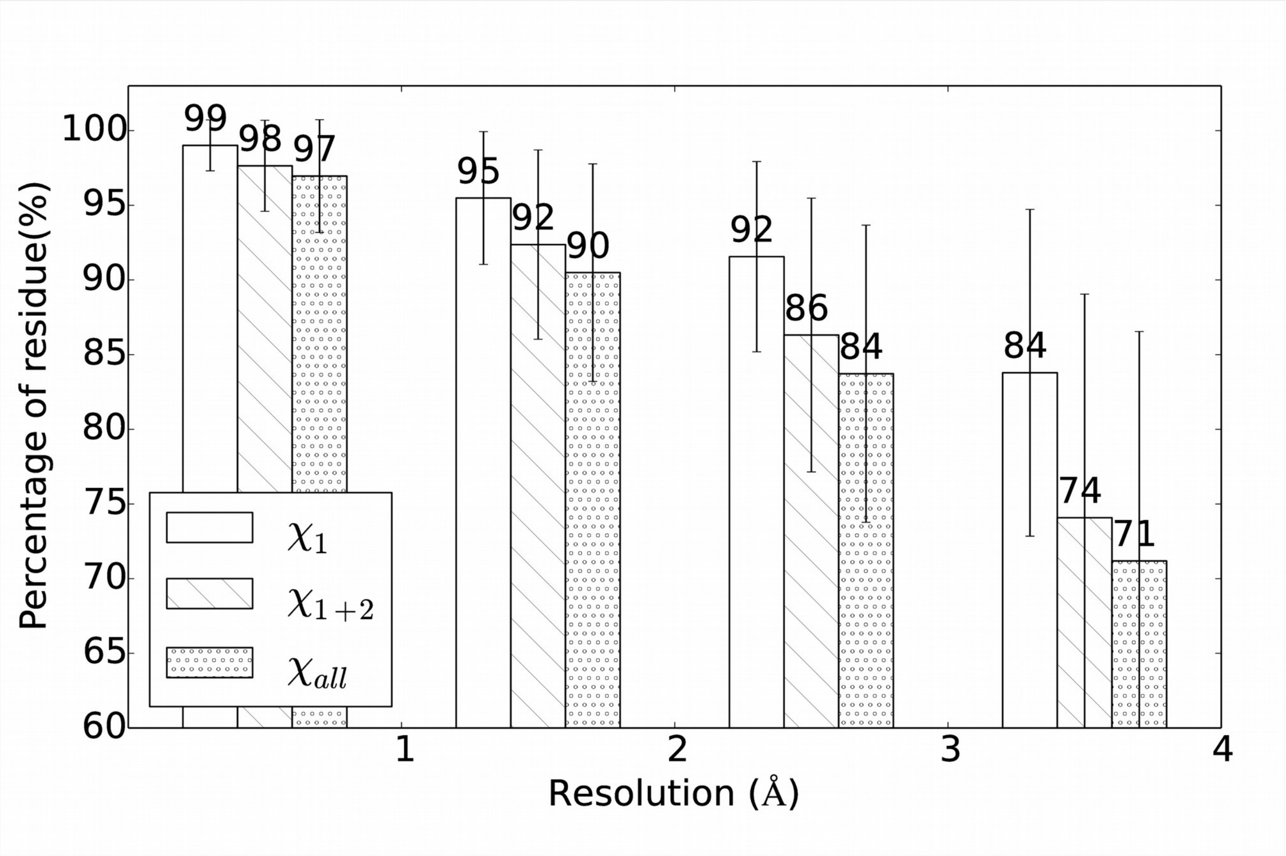

**Fig S10**. Percentages of residues that adopt the same conformation in different crystals of the same protein (set3) as dot plot. Red dots show the residues that keep all the side-chain dihedral conformations, cyan dots show the residues that keep $\chi_1$ and $\chi_2$ dihedrals within 30°, while green dots are residues only keep $\chi_1$.

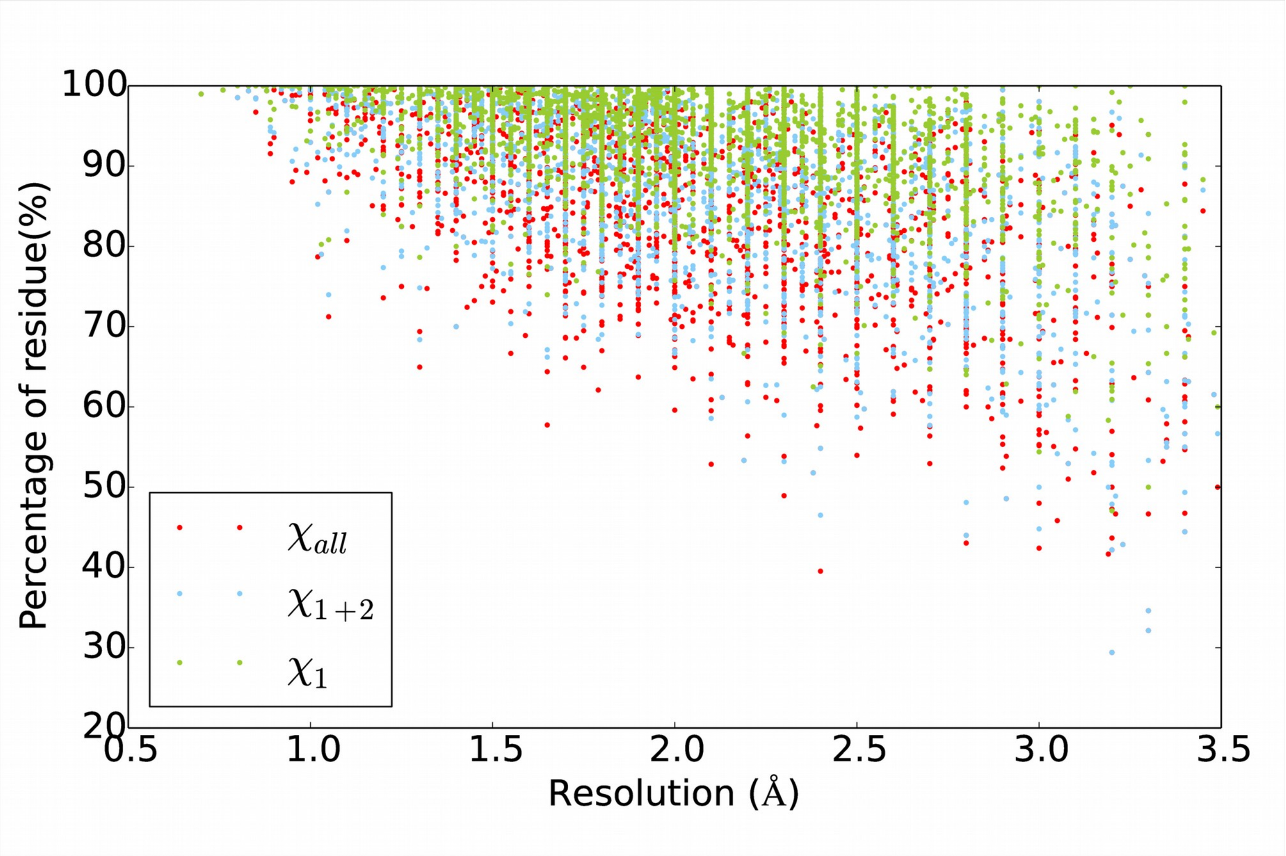

**Fig S11**. Average Percentage of residue keep the same conformation in different crystals of the same protein (set3) counted by residue types.

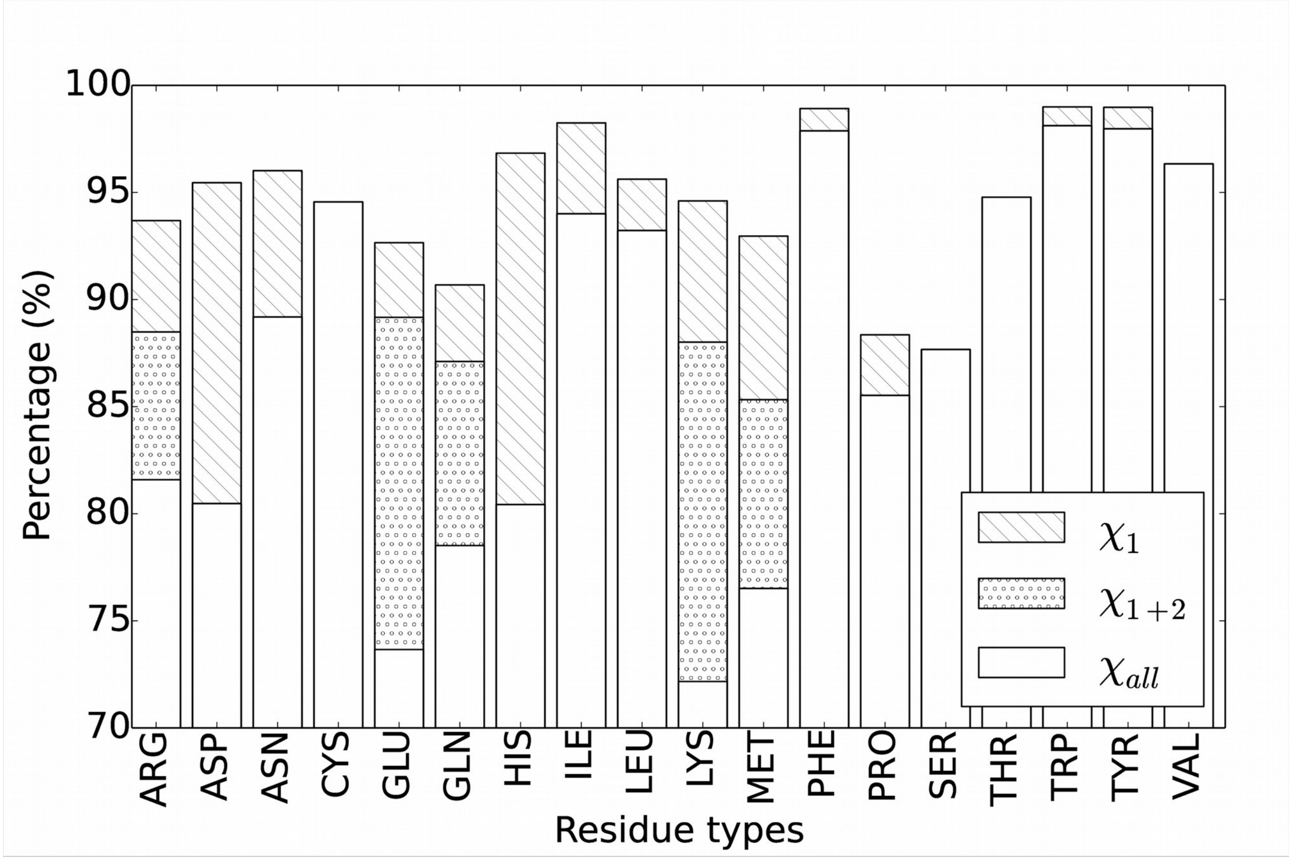

**Fig S12**. Average residue accessible surface area of the residues adopt same conformation (blank bars) or different conformations (dotted) in different crystals of the same protein (set3). Error bars show standard deviations.

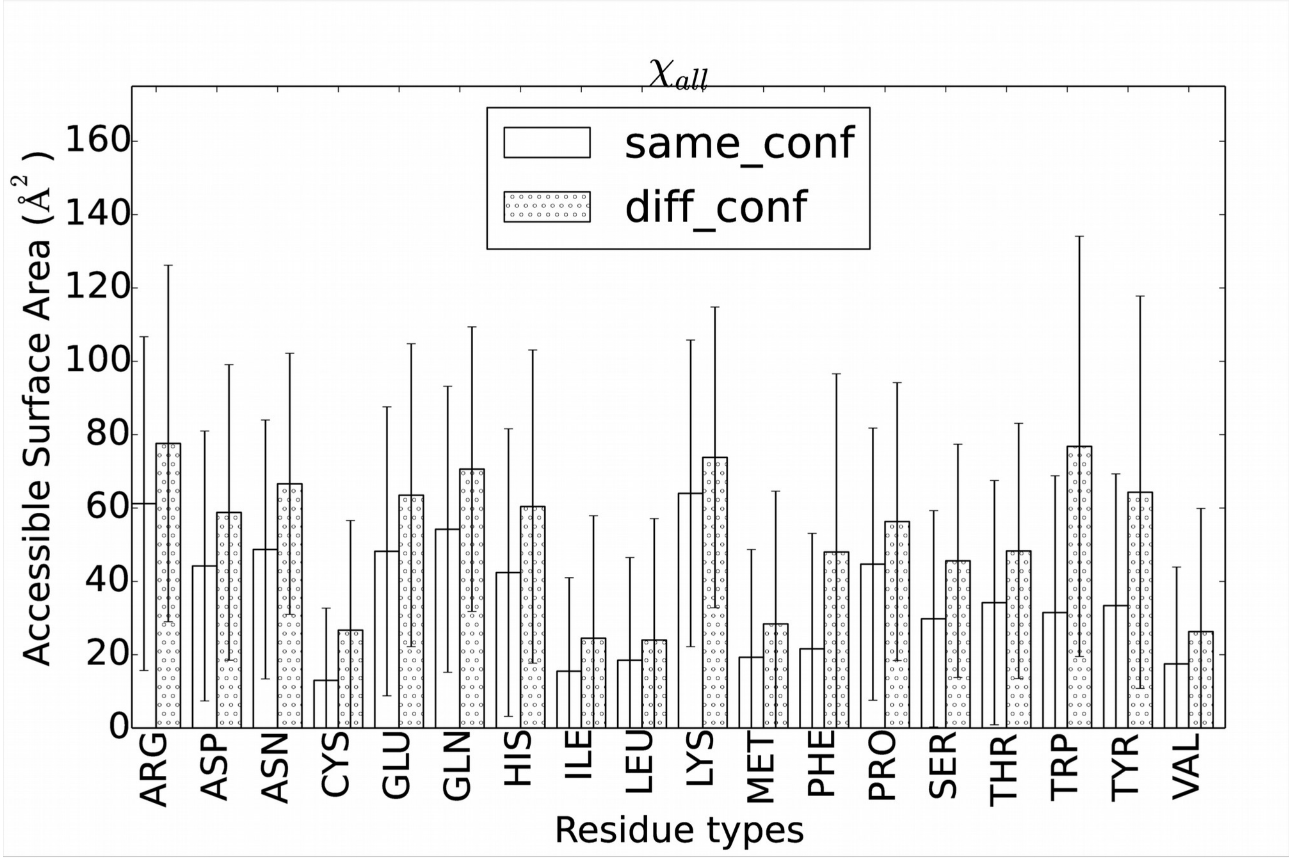

**Fig S13**. Average residue accessible surface area of the residues adopt same $\chi_1$ dihedral (blank bars) or different $\chi_1$ dihedral angles (dotted bars) in different crystals of the same protein (set3). Error bars show standard deviations.

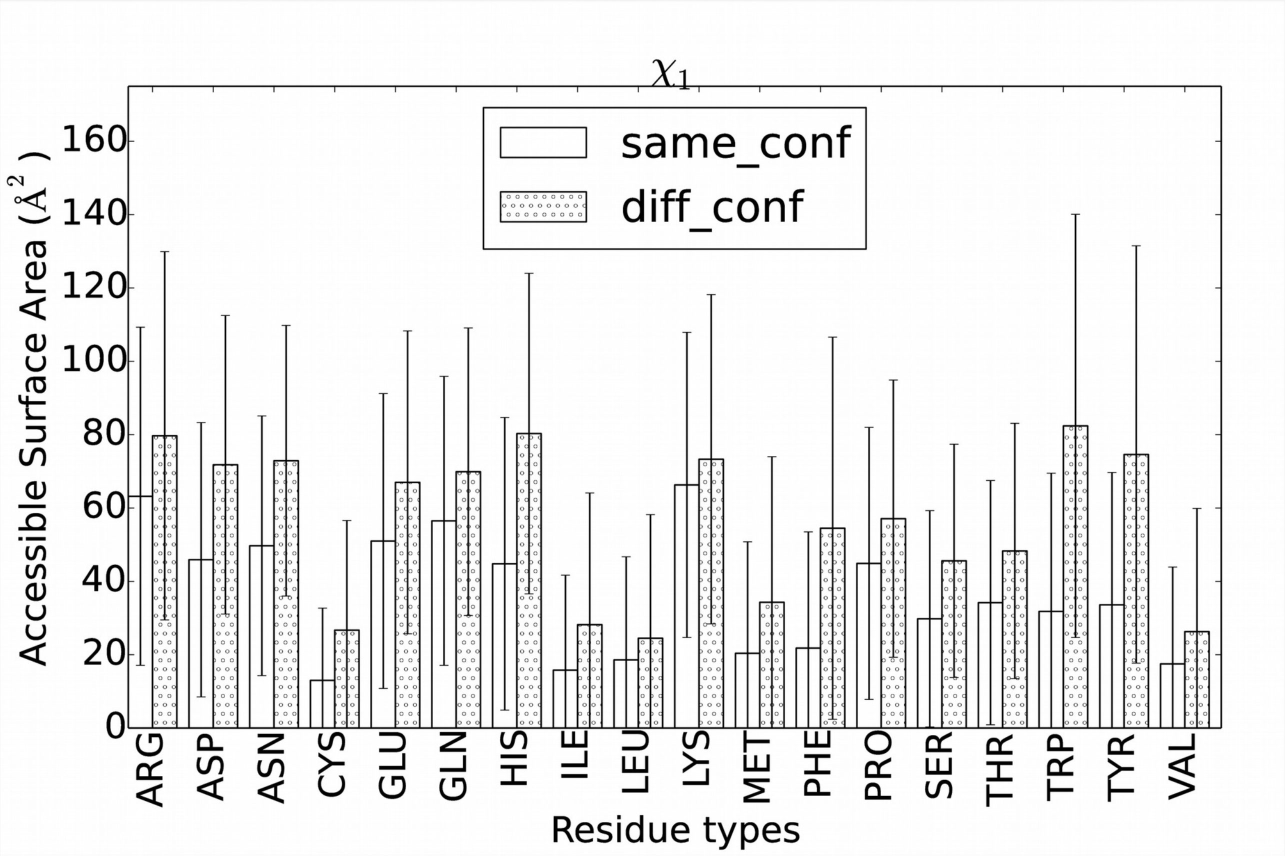

**Fig S14**. Average residue accessible surface area of the residues adopt same $\chi_1$ and $\chi_2$ dihedral (blank bars) or different $\chi_1$ and $\chi_2$ dihedral angles (dotted bars) in different crystals of the same protein (set3). Error bars show standard deviations.

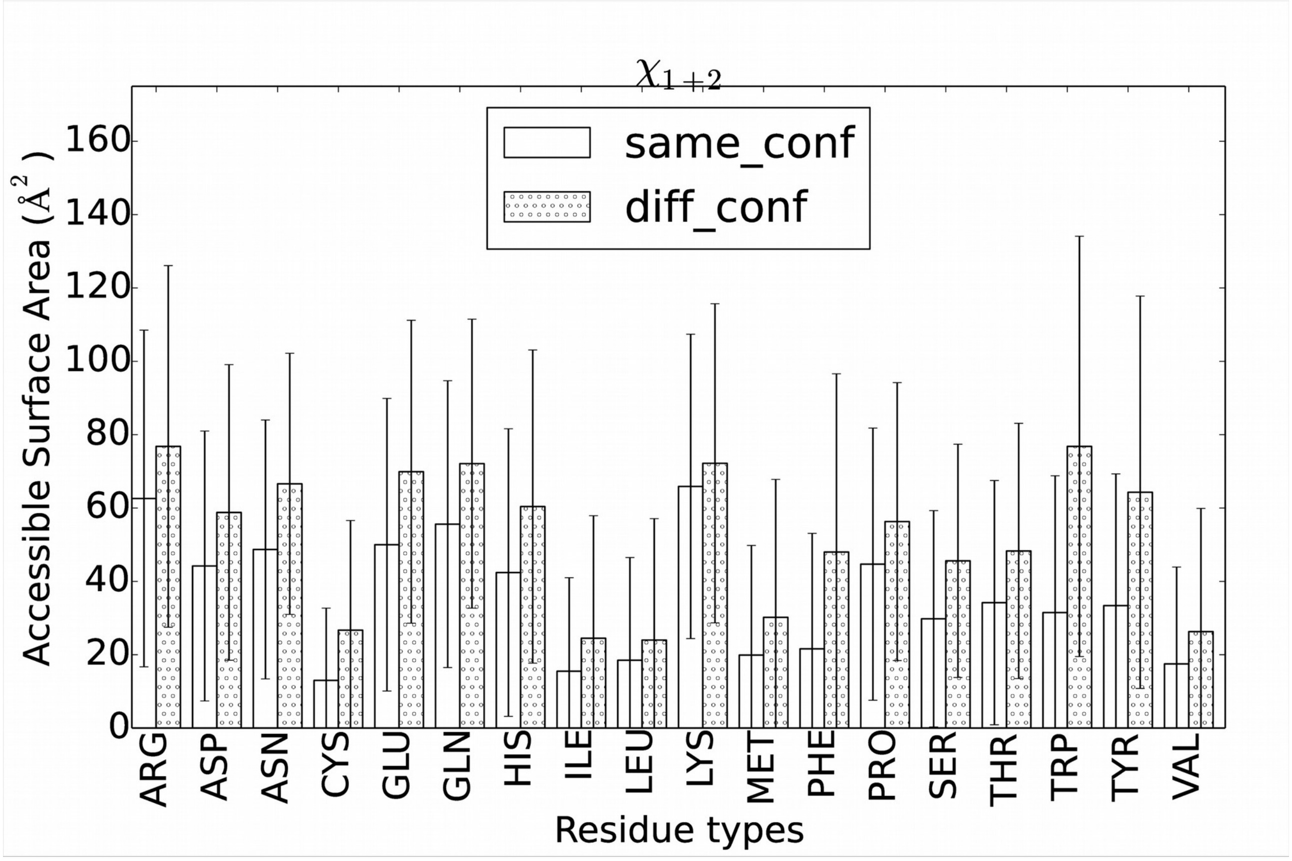

**Fig S15**. Pie plots of residue exposure and conformational change in different chains of the same crystal (set3).

A) Any of the χ dihedrals change is defined as conformational change. Exposed and conformations change amongst alternate location states are shown in red, while same conformations marked in yellow. Buried and same conformations are shown in green, whereas different conformations are shown in blue.

B) $\chi_1$ dihedral change is defined as conformational change, following the same coloring scheme.

C) $\chi_1$ or $\chi_2$ dihedral change is defined as conformational change.

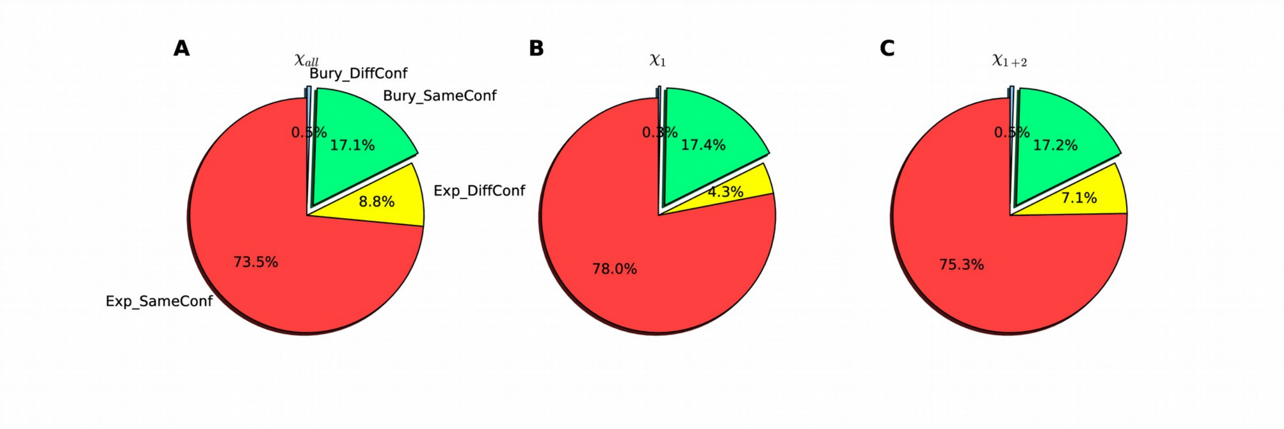

**Fig S16**. Pie plots of residue exposure and conformational change in different crystals of the same protein (set3).

A) Any of the χ dihedrals change is defined as conformational change. Exposed and conformations change amongst alternate location states are shown in red, while same conformations marked in yellow. Buried and same conformations are shown in green, whereas different conformations are shown in blue.

B) $\chi_1$ dihedral change is defined as conformational change, following the same coloring scheme.

C) $\chi_1$ or $\chi_2$ dihedral change is defined as conformational change.

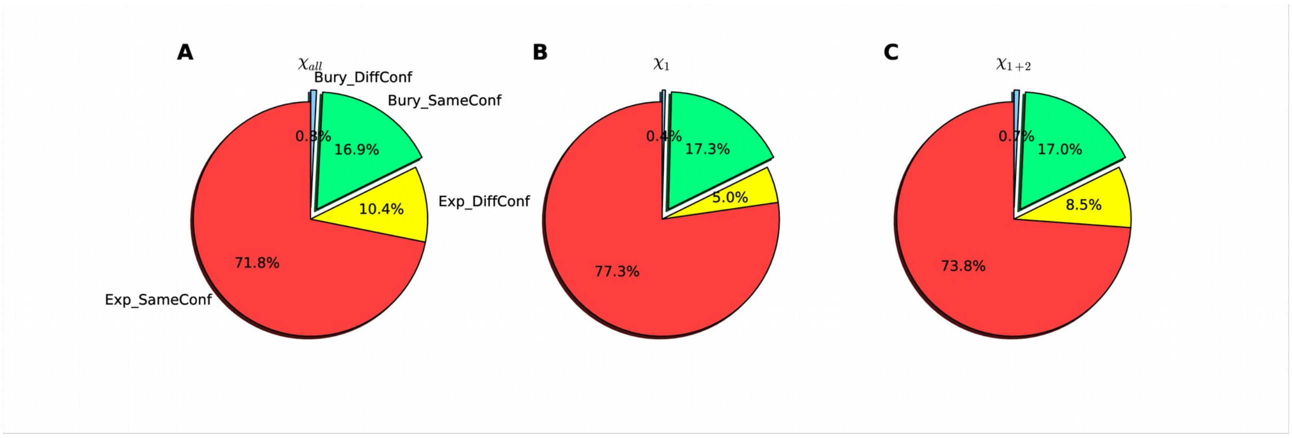

**Fig S17**. Average percentage of residue stay the same conformation in different crystals of the same protein (set3), counted by resolution <1.0Å, 1.0-2.0Å, 2.0-3.0Å and >3.0Å. The blue parts show the residues that adopt the same backbone conformation, others are all residue counts.

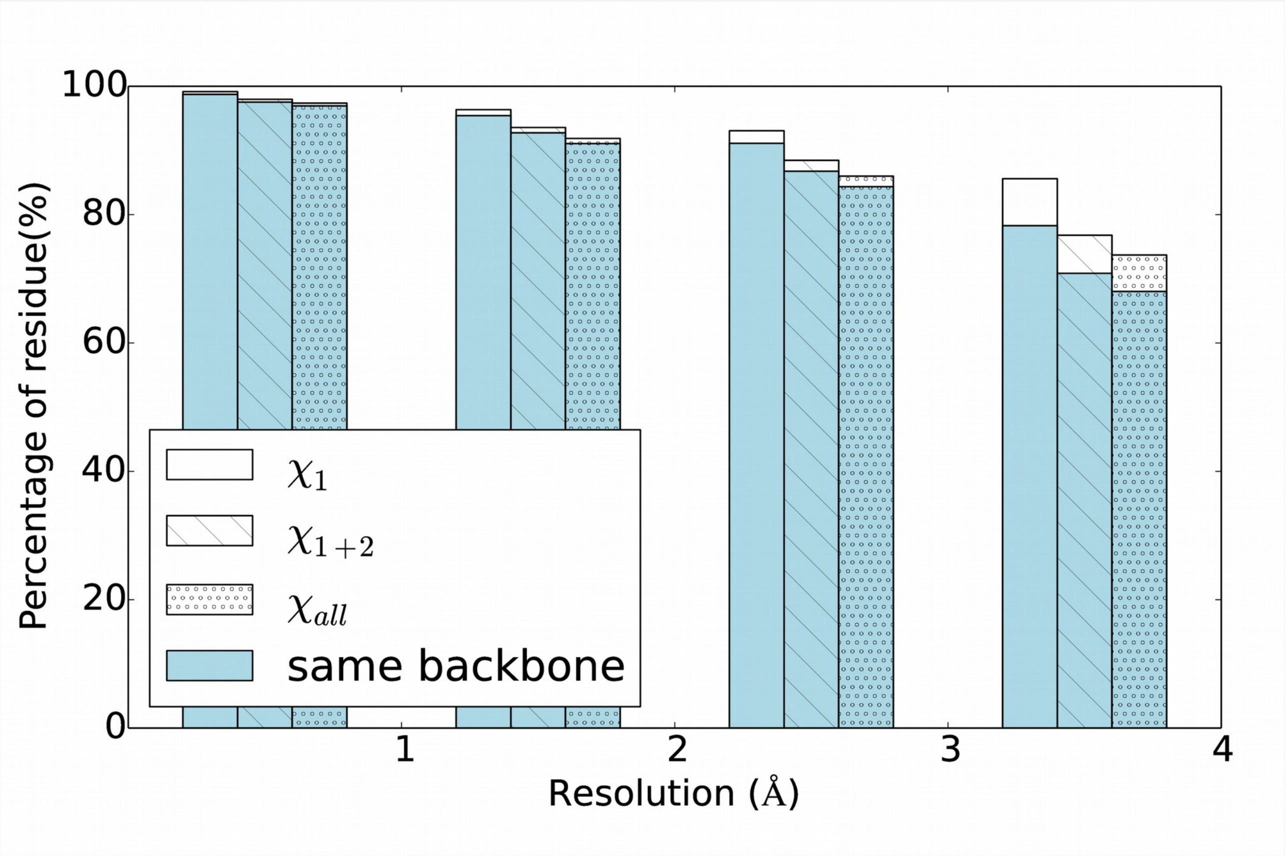

**Fig S18**. Average Percentage of residue keep the same conformation in different crystals of the same protein (set3) counted by residue types, while the blue parts show the residues with the same backbone conformations.

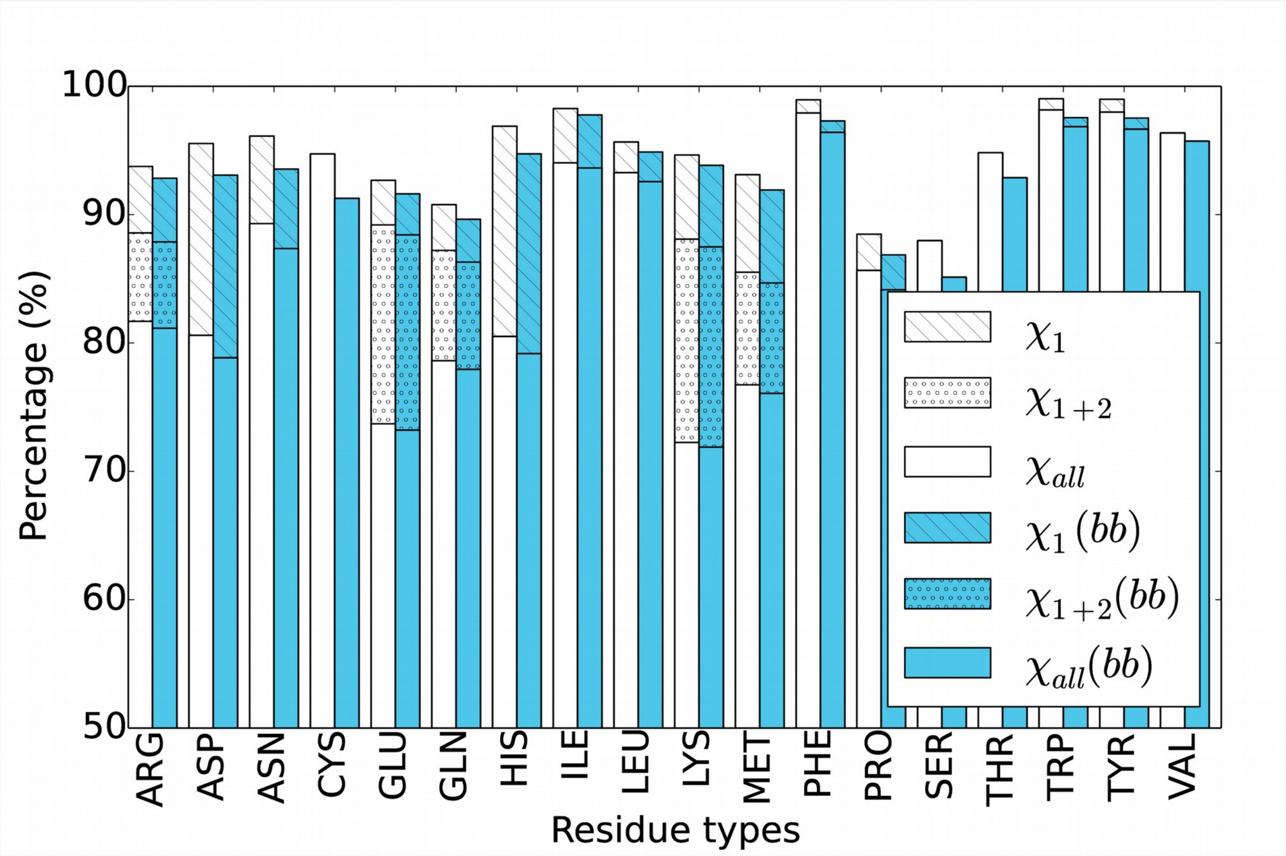

**Fig S19**. Average percentage of residue stay the same conformation in different crystals of the same protein (set4), counted by resolution <1.0Å, 1.0-2.0Å, 2.0-3.0Å and >3.0Å. Error bars show standard deviations.

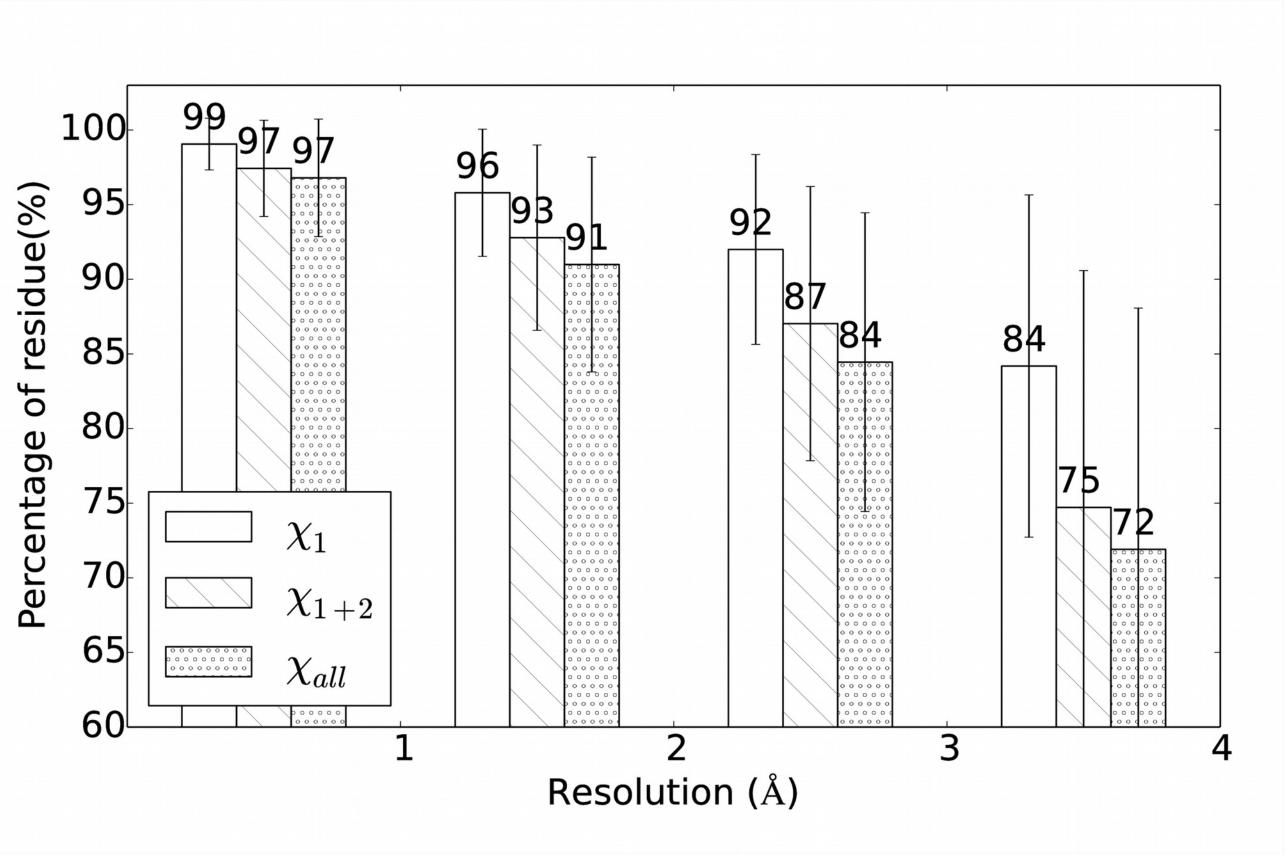

**Fig S20**. Percentages of residues that adopt the same conformation in different crystals of the same protein (set4) as dot plot. Red dots show the residues that keep all the side-chain dihedral conformations, cyan dots show the residues that keep $\chi_1$ and $\chi_2$ dihedrals within 30°, while green dots are residues only keep $\chi_1$.

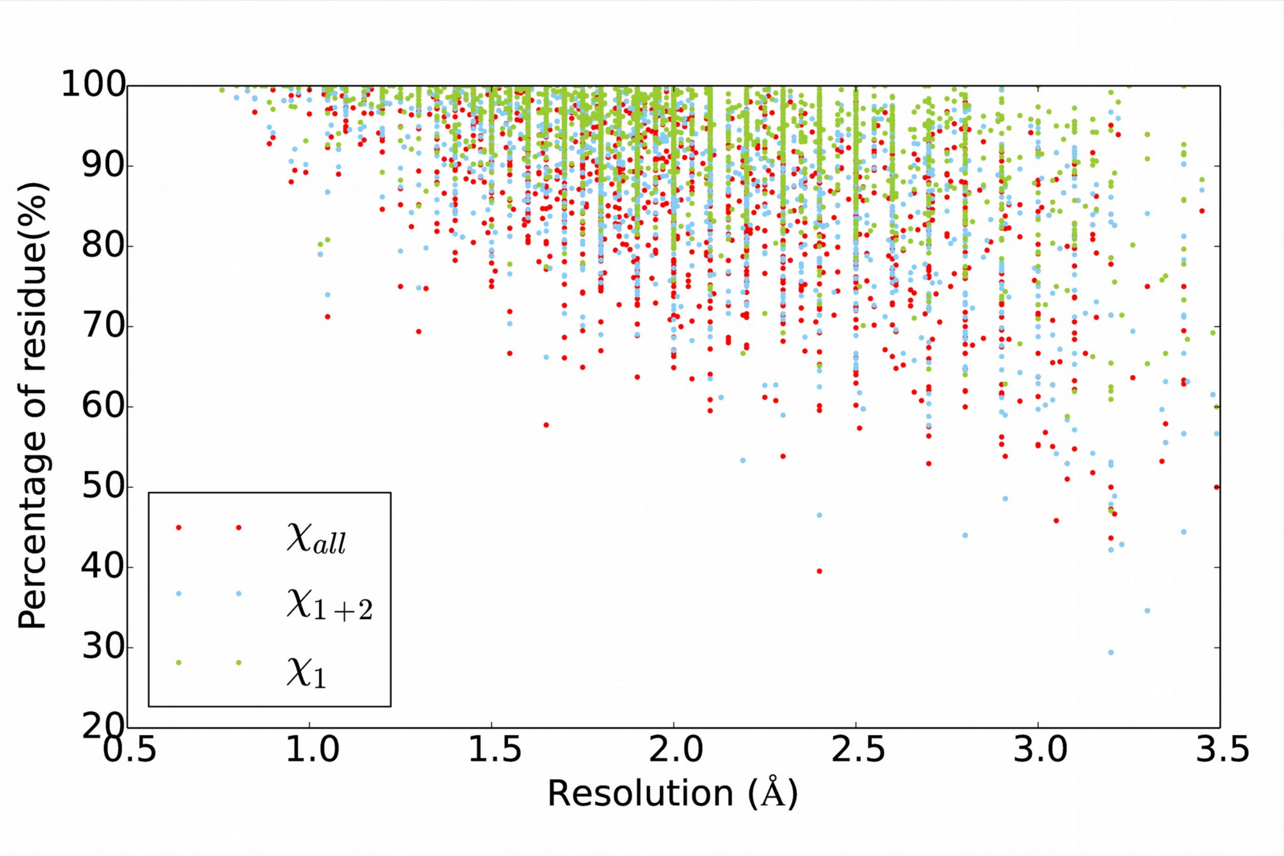

**Fig S21**. Average Percentage of residue keep the same conformation in different crystals of the same protein (set4) counted by residue types.

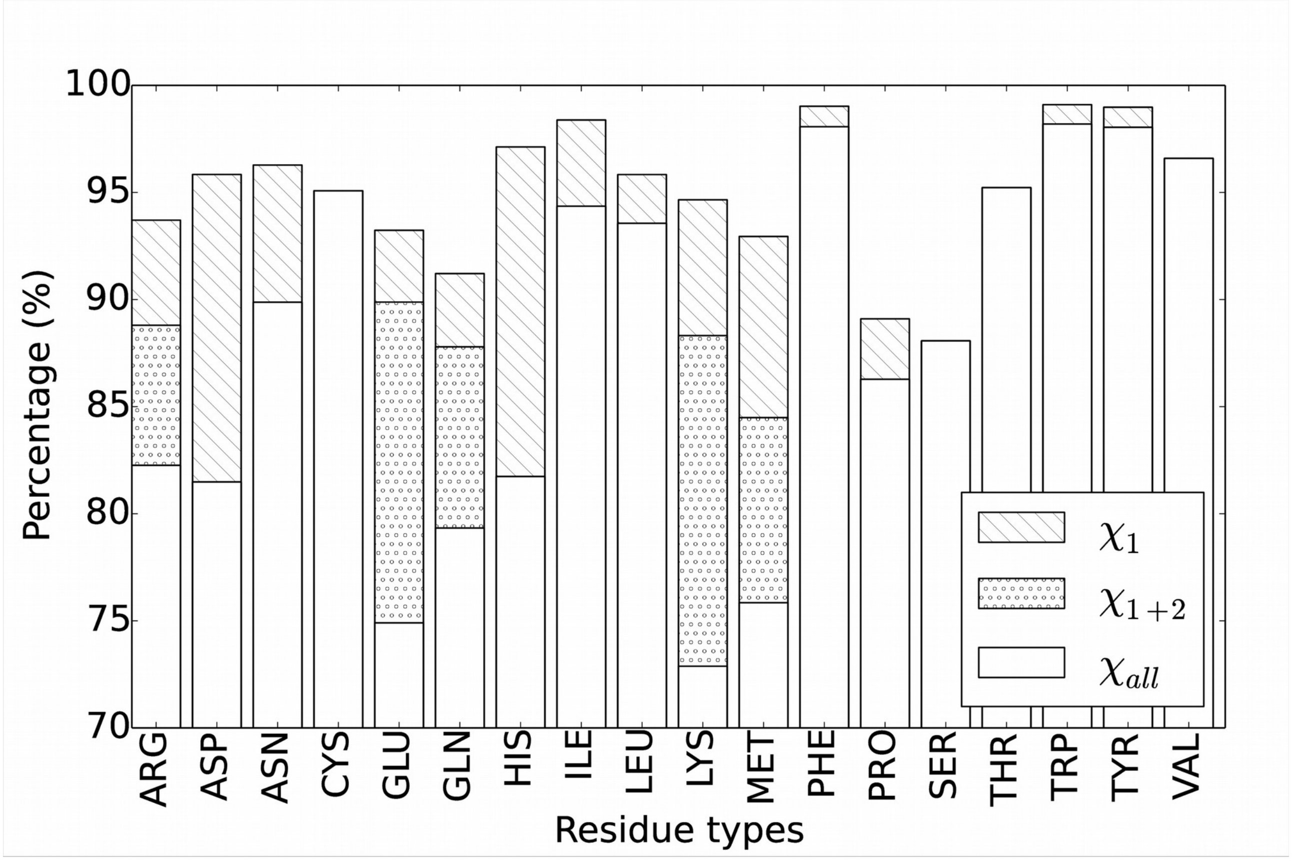

**Fig S22**. Average residue accessible surface area of the residues adopt same conformation (blank bars) or different conformations (dotted bars) in different crystals of the same protein (set4). Error bars show the standard deviations.

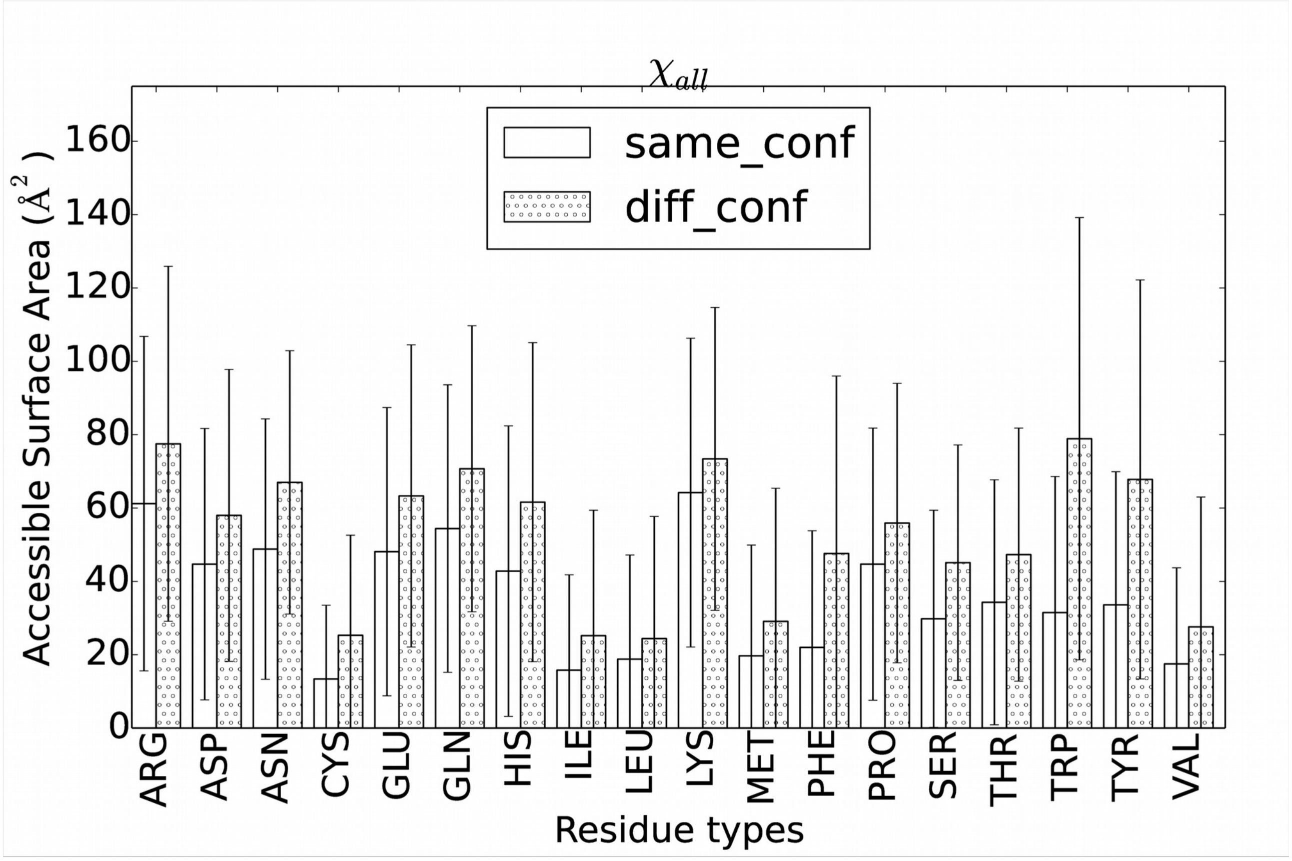

**Fig S23**. Average residue accessible surface area of the residues adopt same $\chi_1$ dihedral (blank bars) or different $\chi_1$ dihedral angles (dotted bars) in different crystals (set4). Error bars show the standard deviations.

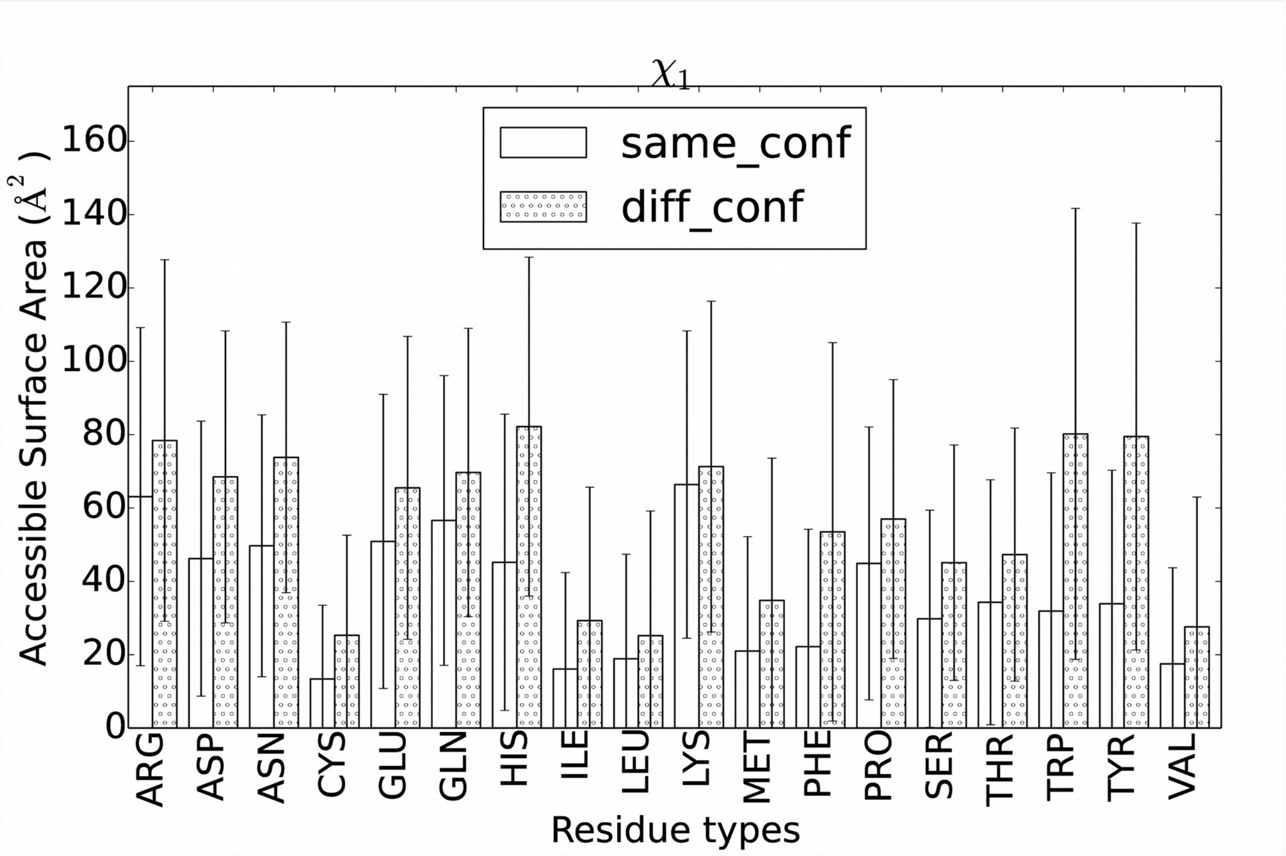

**Fig S24**. Average residue accessible surface area of the residues adopt same $\chi_1$ and $\chi_2$ dihedral or different $\chi_1$ and $\chi_2$ dihedral angles in different crystals (set4).

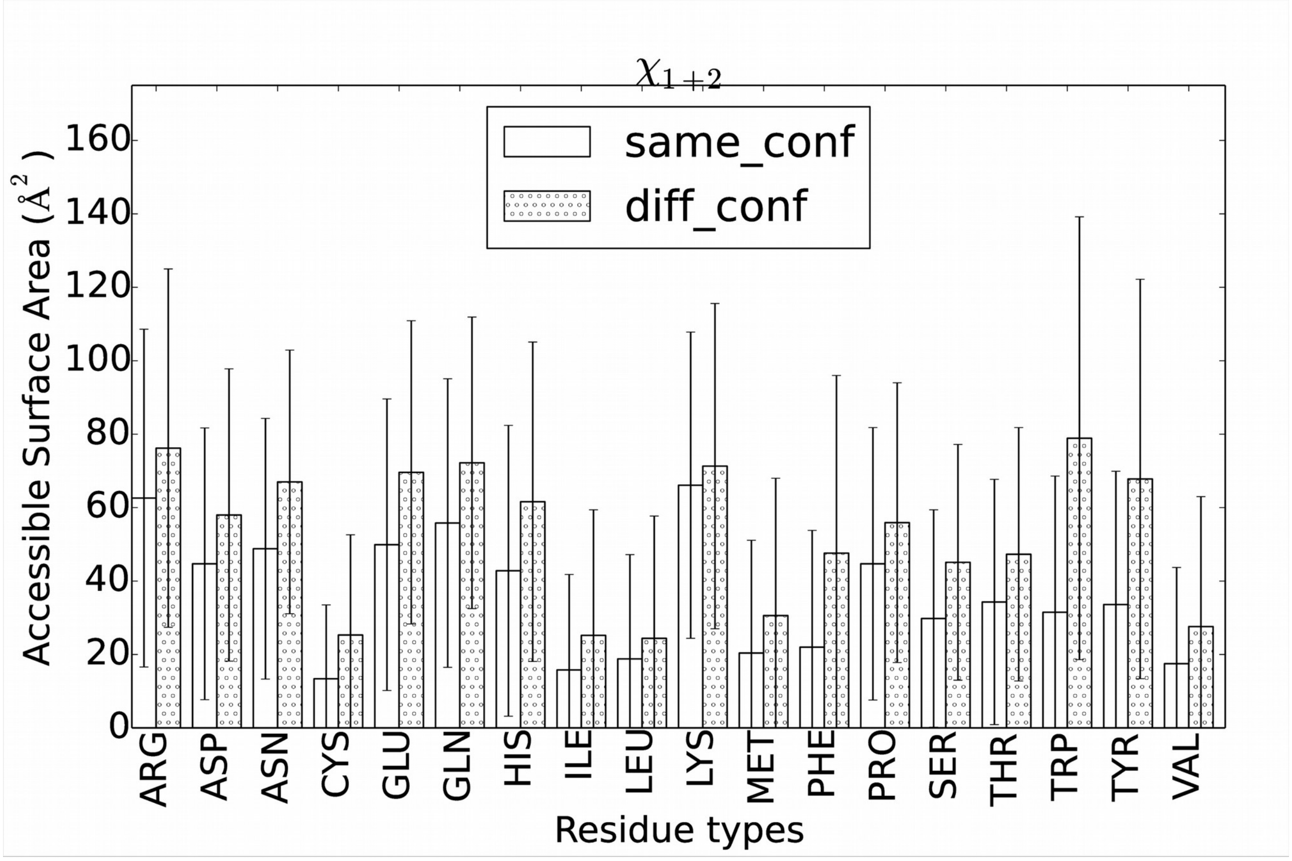

**Fig S25**. Average percentage of residue stay the same conformation in different crystals of the same protein (set4), counted by resolution <1.0Å, 1.0-2.0Å, 2.0-3.0Å and >3.0Å. The blue parts show the residues that adopt the same backbone conformation.

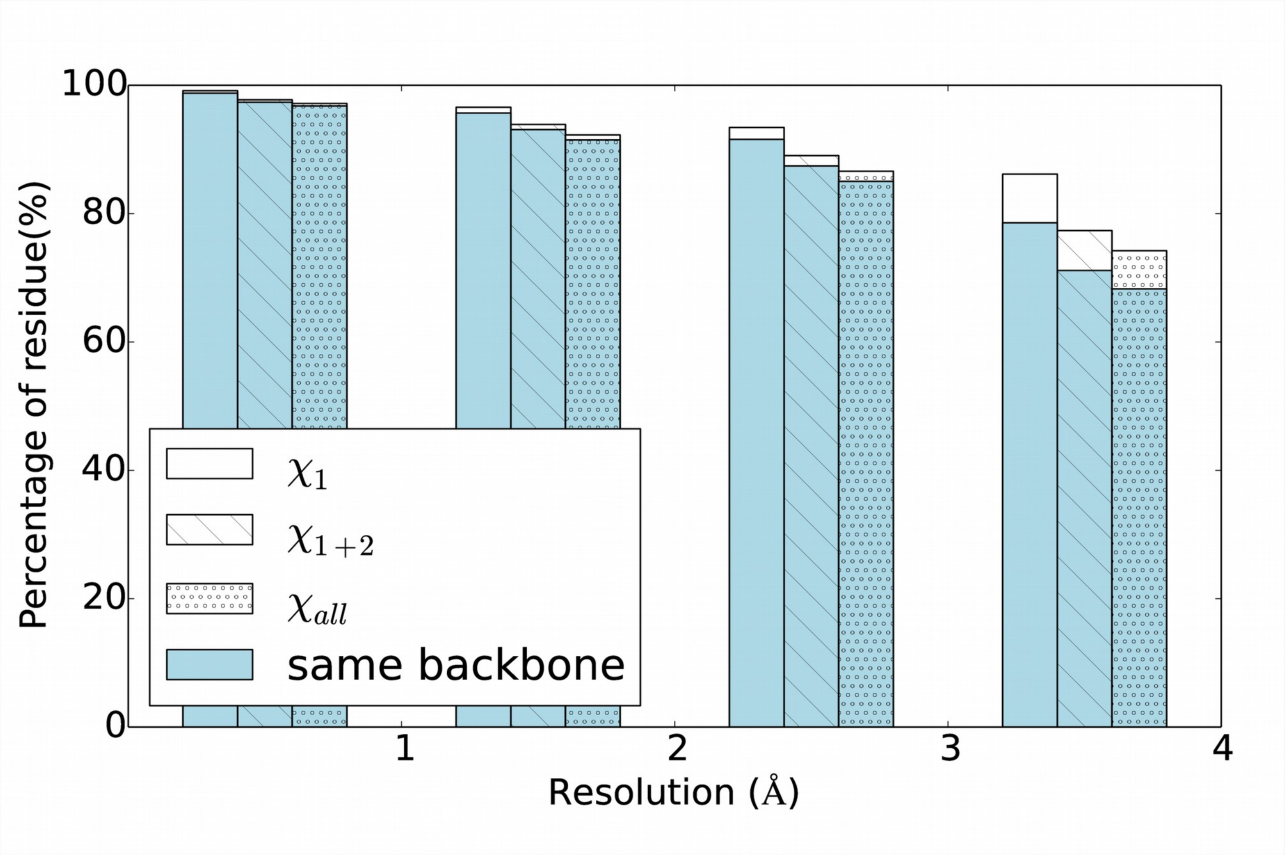

**Fig S26**. Average Percentage of residue keep the same conformation in different crystals of the same protein (set4) counted by residue types, while the blue parts show the residues with different backbone conformations.

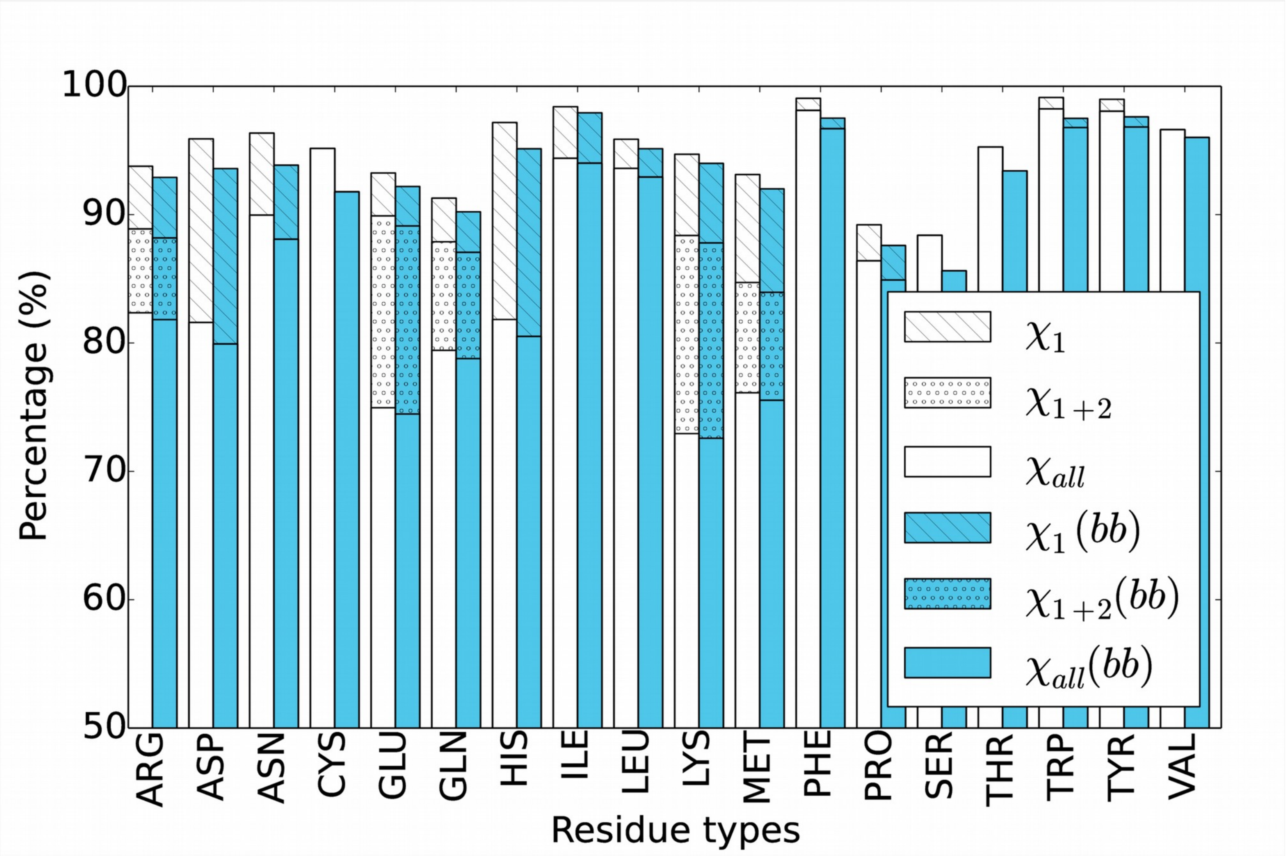

**Fig S27**. Pie plots of residue exposure and conformational change in different crystals of the same protein (set4).

A) Any of the χ dihedrals change is defined as conformational change. Exposed and conformations change amongst alternate location states are shown in red, while same conformations marked in yellow. Buried and same conformations are shown in green, whereas different conformations are shown in blue.

B) $\chi_1$ dihedral change is defined as conformational change, following the same coloring scheme.

C) $\chi_1$ or $\chi_2$ dihedral change is defined as conformational change.

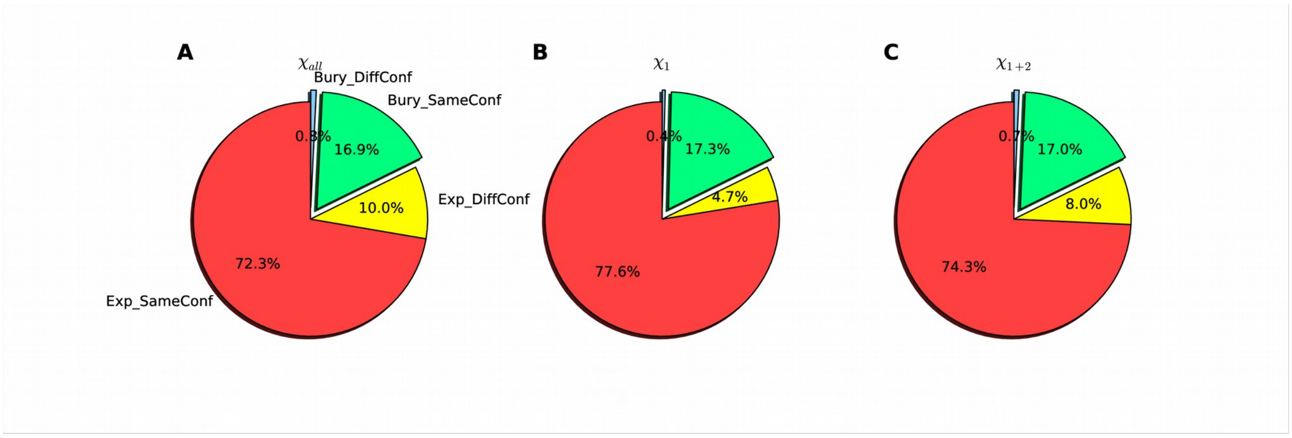

**Fig S28**. Average percentage of residue stay the same conformation in different crystals of the same protein (set5), counted by resolution <1.0Å, 1.0-2.0Å, 2.0-3.0Å and >3.0Å. Error bars show standard deviations.

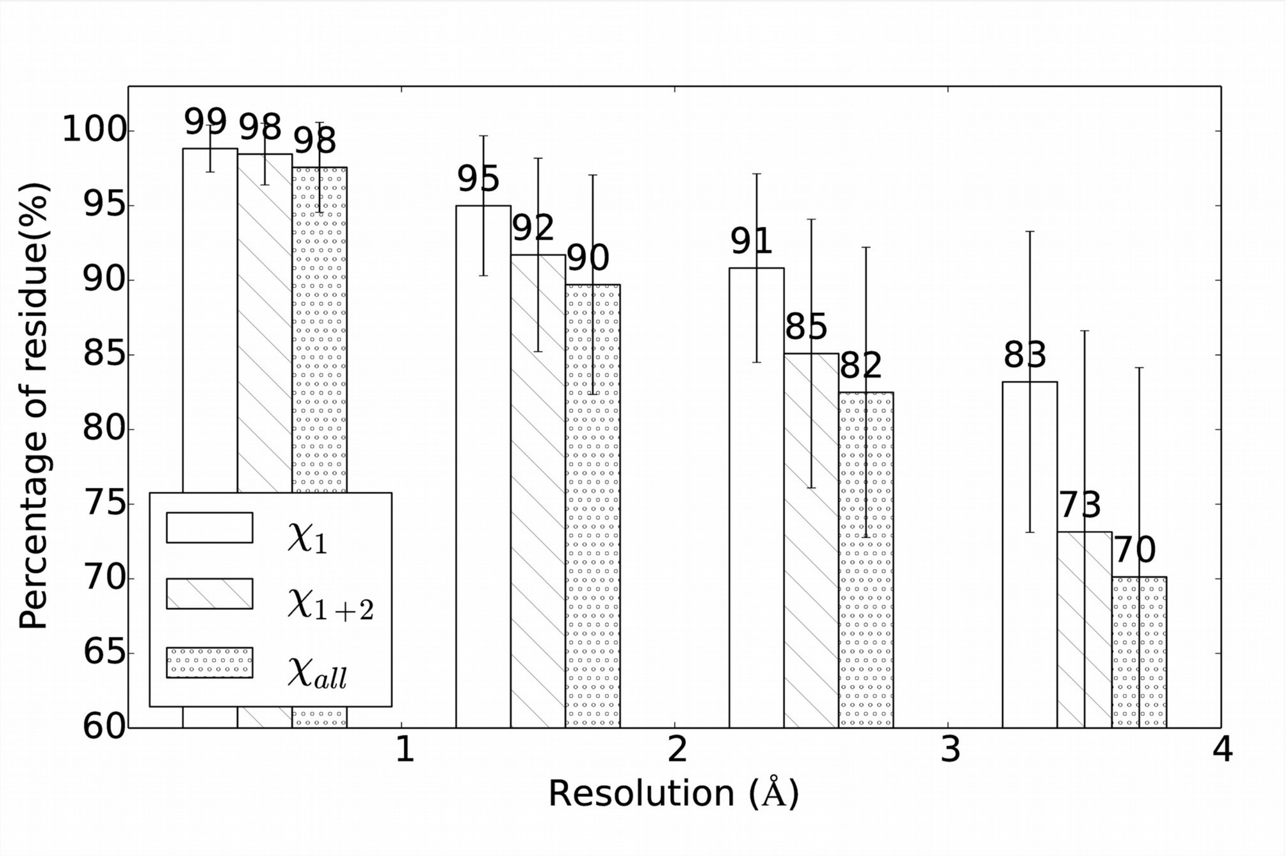

**Fig S29**. Percentages of residues that adopt the same conformation in different crystals of the same protein (set5) as dot plot. Red dots show the residues that keep all the side-chain dihedral conformations, cyan dots show the residues that keep $\chi_1$ and $\chi_2$ dihedrals within 30°, while green dots are residues only keep $\chi_1$.

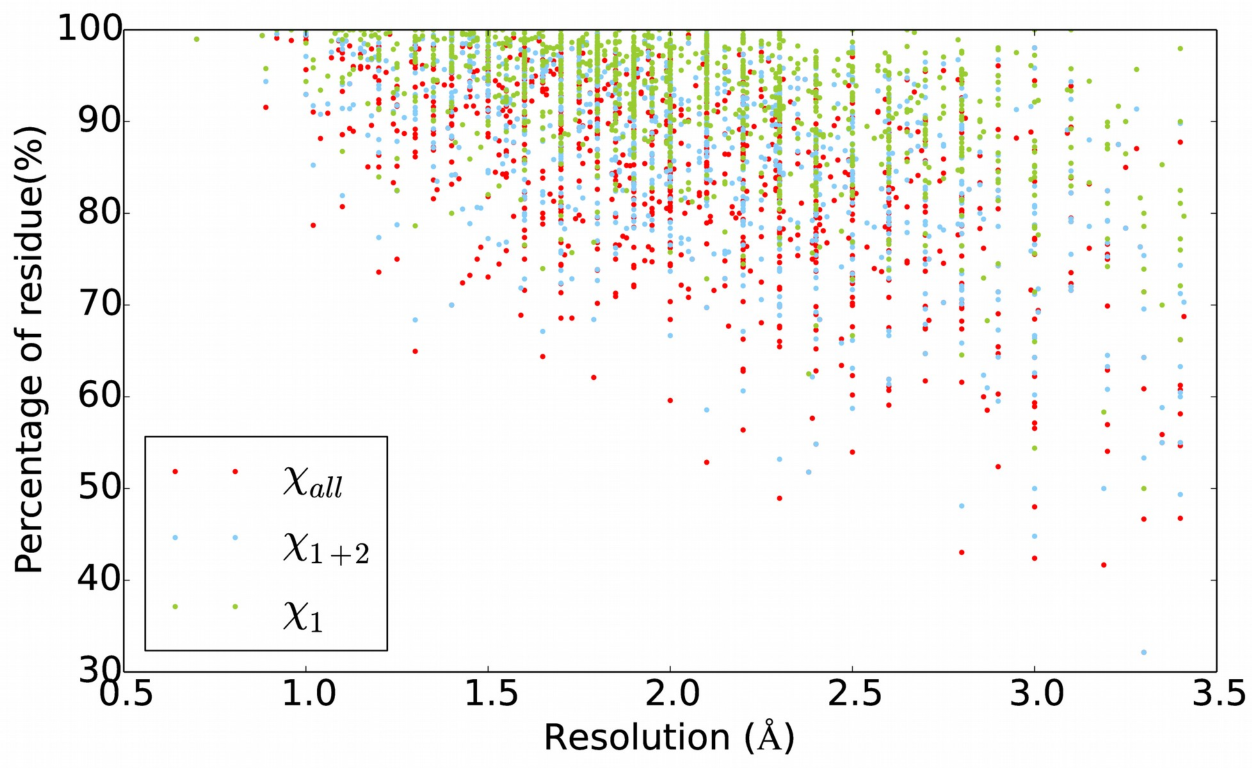

**Fig S30**. Average Percentage of residue keep the same conformation in different crystals of the same protein (set5) counted by residue types.

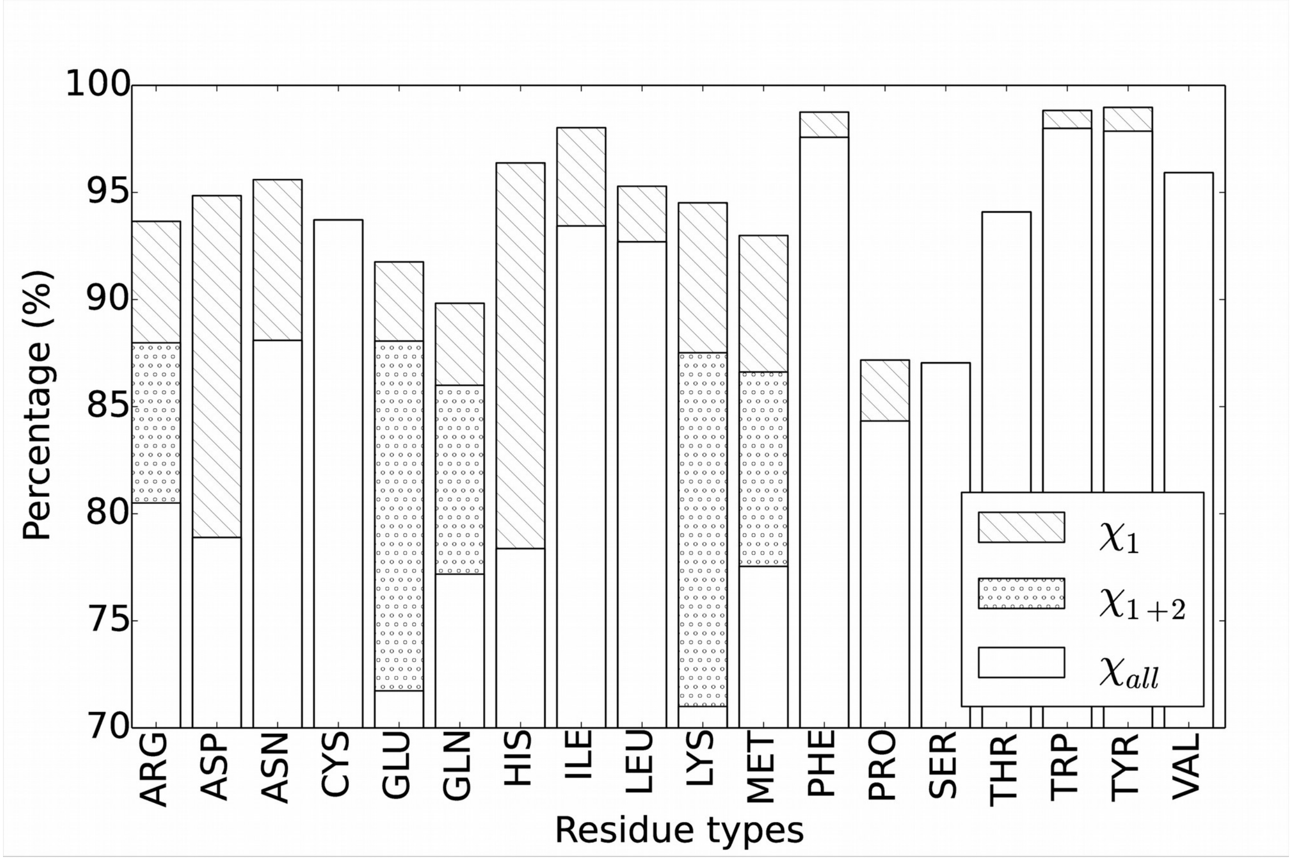

**Fig S31**. Average residue accessible surface area of the residues adopt same conformation (blank bars) or different conformations (dotted bars) in different crystals of the same protein (set5). Error bars show the standard deviations.

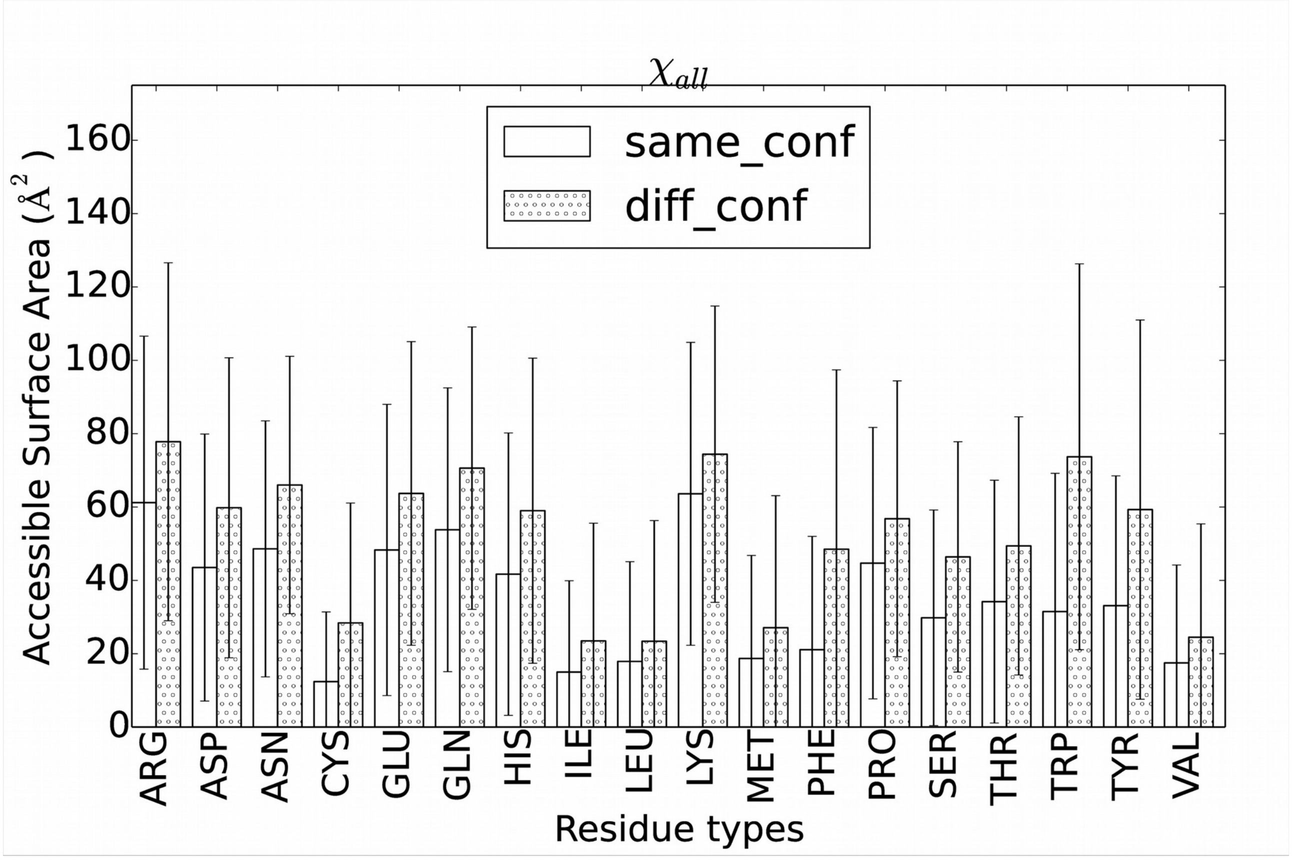

**Fig S32**. Average residue accessible surface area of the residues adopt same $\chi_1$ dihedral (blank bars) or different $\chi_1$ dihedral angles (dotted bars) in different crystals (set5). Error bars show the standard deviations.

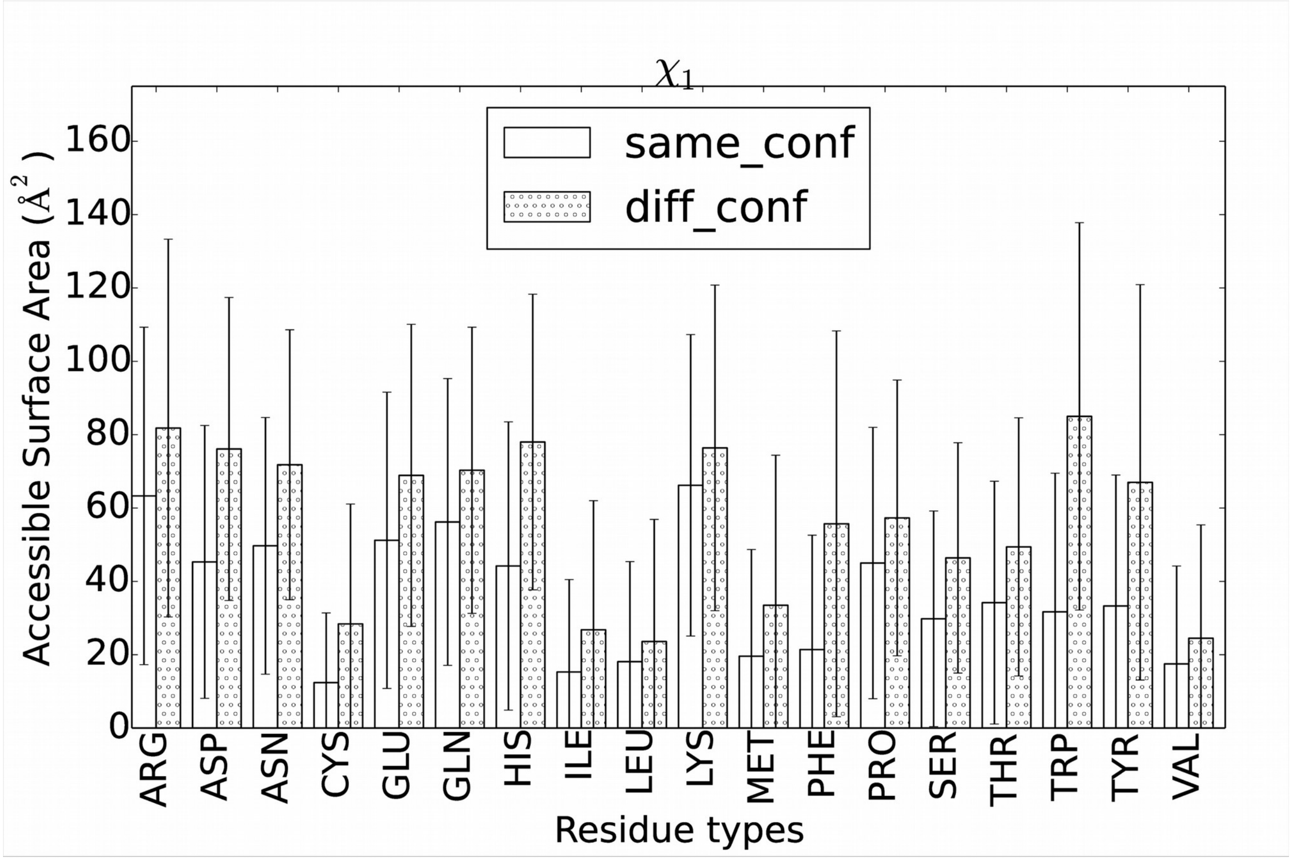

**Fig S33**. Average residue accessible surface area of the residues adopt same $\chi_1$ and $\chi_2$ dihedral or different $\chi_1$ and $\chi_2$ dihedral angles in different crystals (set5).

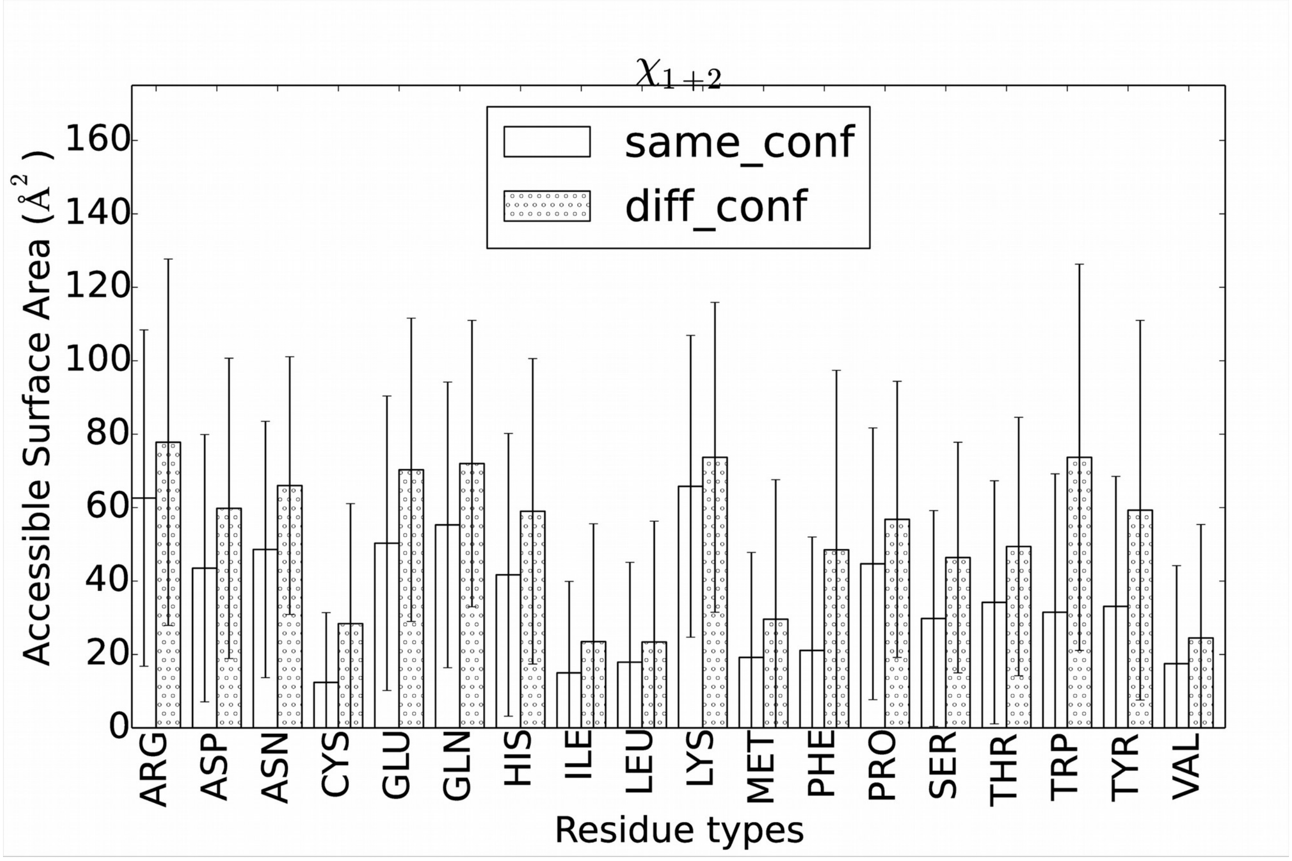

**Fig S34**. Average percentage of residue stay the same conformation in different crystals of the same protein (set5), counted by resolution <1.0Å, 1.0-2.0Å, 2.0-3.0Å and >3.0Å. The blue parts show the residues that adopt the same backbone conformation.

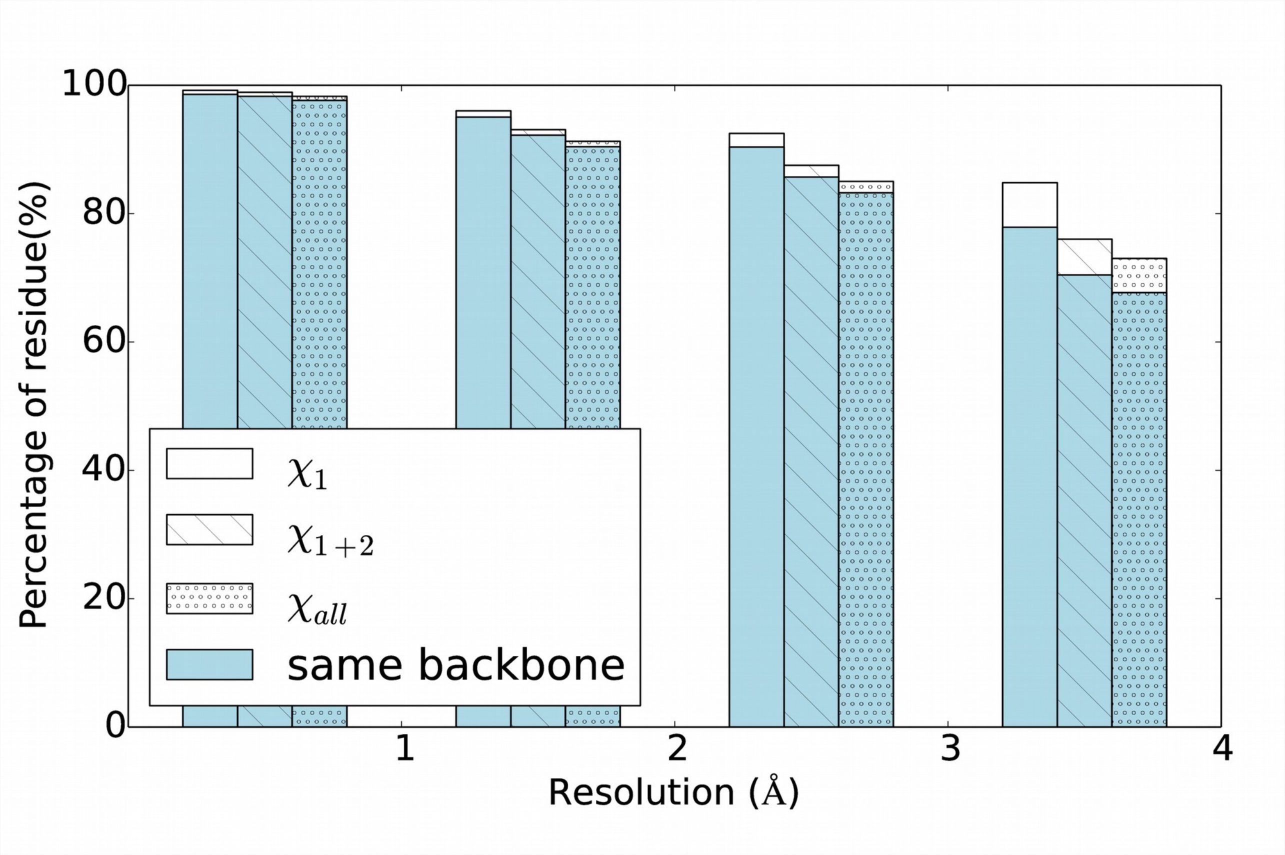

**Fig S35**. Average Percentage of residue keep the same conformation in different crystals of the same protein (set5) counted by residue types, while the blue parts show the residues with different backbone conformations.

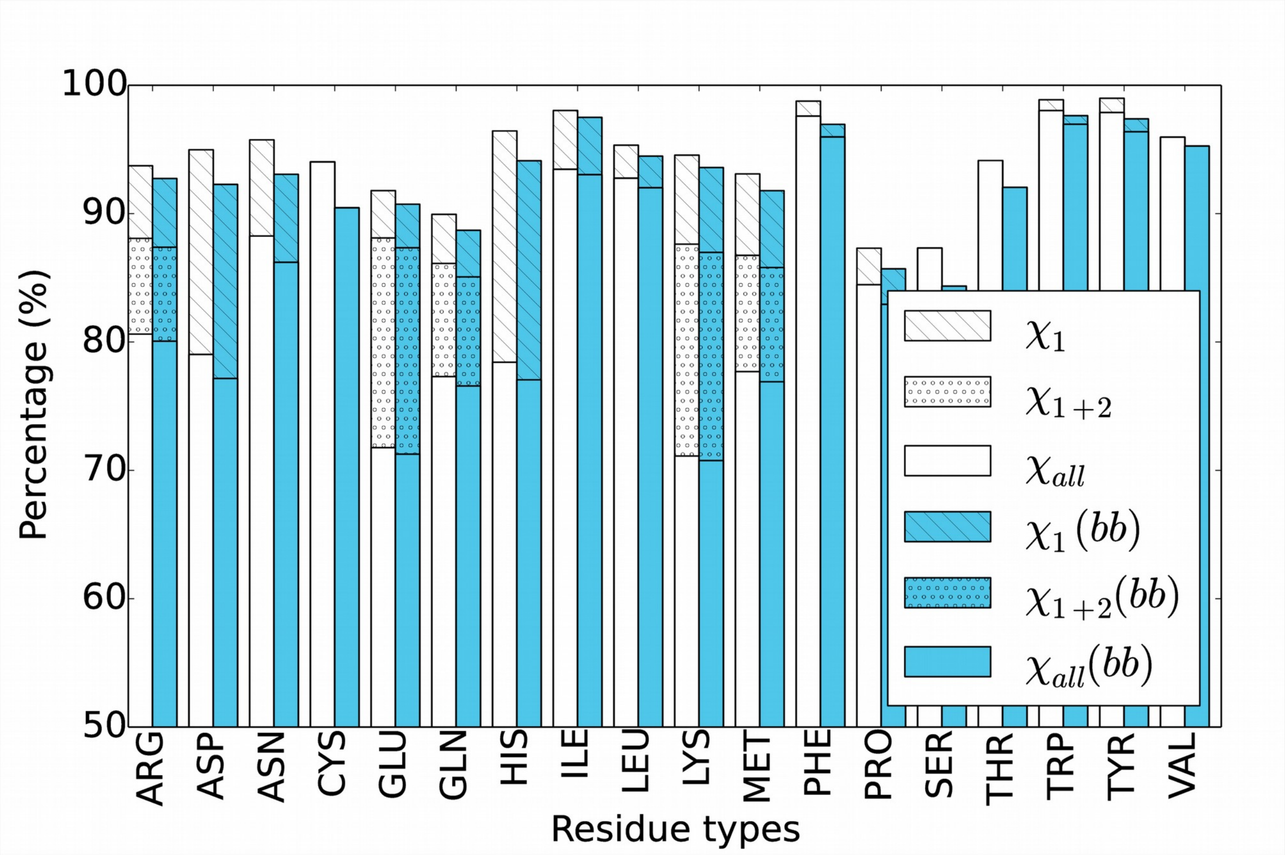

**Fig S36**. Pie plots of residue exposure and conformational change in different crystals of the same protein (set5).

A) Any of the $\chi$ dihedrals change is defined as conformational change. Exposed and conformations change amongst alternate location states are shown in red, while same conformations marked in yellow. Buried and same conformations are shown in green, whereas different conformations are shown in blue.

B) $\chi_1$ dihedral change is defined as conformational change, following the same coloring scheme.

C) $\chi_1$ or $\chi_2$ dihedral change is defined as conformational change.

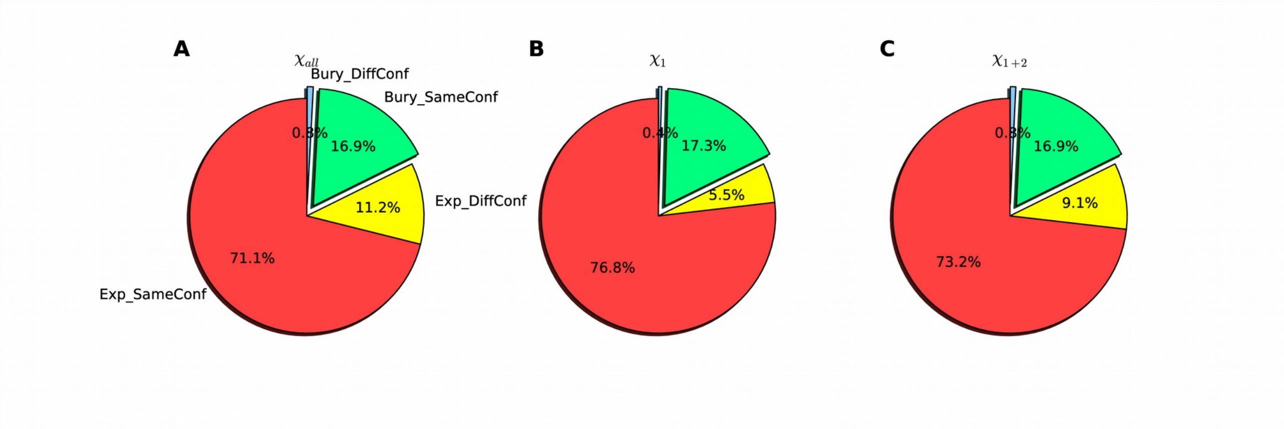

**Fig S37**. General Residue accessible surface area distribution of Residues with and without alternate location.

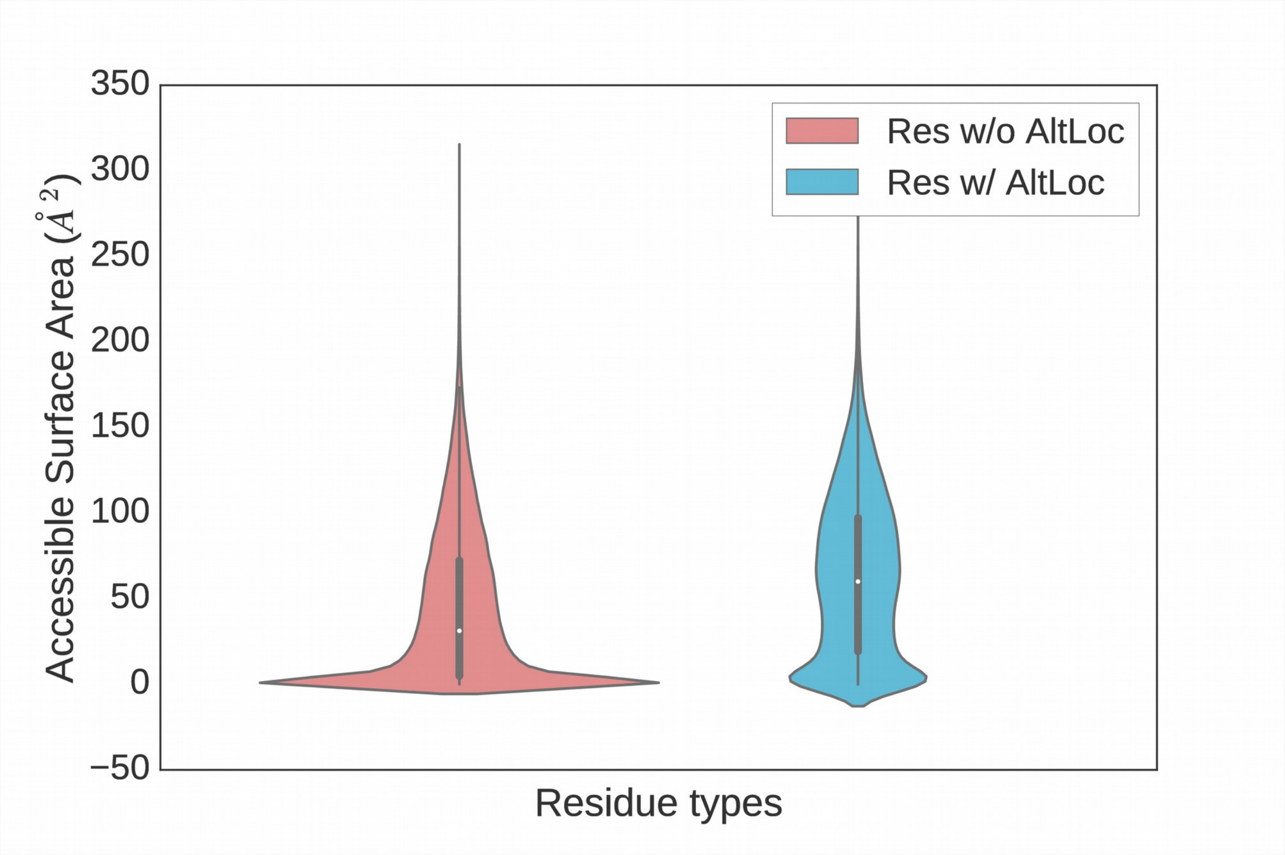

**Table Legends**

**Table S1**. List of dataset set1. The three columns are: PDB id, resolution and length of the protein.

**Table S2**. List of reliable atoms/residue counts in each protein. The four columns of the table are: PDB id, resolution, percentage of reliable atoms and percentage of reliable residues.

**Table S3**. Unreliable atoms/residue counts by residue type. For each residue, we count average number of unreliable atom per residue, the standard deviation, percentage of unreliable residue in total count of this type of residue and total counts of this type of residue.

**Table S4.** Average residue accessible surface area of reliable and unreliable residues (set1). The five columns of the table are: residue type, average residue accessible surface area (RSA) of residues with clear electron density, standard deviation of this count, average RSA of residues with unclear electron density, standard deviation. Corresponding to Fig 2D.

**Table S5**. List of reliable side-chain atoms counts in each protein. The four columns are: PDB id, resolution, percentage of reliable atoms of the structure and percentage of reliable residues of the structure.

**Table S6**. Unreliable side-chain atom counts by residue type. The five columns are: residue type, average unreliable atom per residue, standard deviation, percentage of unreliable residues in total count of this type of residue and total counts of this type of residue.

**Table S7.** Average side-chain accessible surface area of reliable and unreliable residues. The five columns of the table are: residue type, average residue accessible surface area (RSA) of residues with clear electron density, standard deviation of this count, average RSA of residues with unclear electron density and standard deviation. Corresponding to Fig S2C.

**Table S8**. Alternate location residue number counts by protein. The four columns are PDB id, number of residues with alternate locations, length of the protein and resolution. (Proteins in set1 with more than one alternate location residue.)

**Table S9**. Alternate location residue number counts by residue type. The columns are: residue type, total number of counts and number of residue with alternate locations.

**Table S10**. Average residue accessible surface area of residues with and without alternate location atoms. The five columns are: residue type, average RSA of residues without alternate locations, standard deviation, average RSA of residues with alternate locations and standard deviation.

**Table S11**. The list of protein chain pairs from the same crystal, dataset set2. Reso1 and Reso2 are resolutions of the two structures, Len1 and Len2 are lengths of the two structures, SeqId is sequence identity measured by sequence alignment, while SeqID_aligned is sequence identity of the structurally aligned part.

**Table S12**. Number of residues that keep the conformation counted by protein. The seven columns of the table are: PDB id, PDB id, number of residues keep same $\chi_1$ conformation, number of residues keep same $\chi_{1+2}$ conformations, number of residues keep same $\chi_{all}$ conformations, total number of aligned residue and resolution of the structure.

**Table S13**. Number of residues that keep the conformation for $\chi_{1,}$ $\chi_{1+2}$, $\chi_{all}$ counted by residue type. The last column is total number of counts.

**Table S14**. The list of protein chain pairs from the different crystal, dataset set3. Reso1 and Reso2 are resolutions of the two structures, Len1 and Len2 are lengths of the two structures, SeqId is sequence identity measured by sequence alignment, while SeqID_aligned is sequence identity of the structurally aligned part.

**Table S15**. The list of protein chain pairs from the different crystal with 100% sequence identity, dataset set4. Reso1 and Reso2 are resolutions of the two structures, Len1 and Len2 are lengths of the two structures, SeqId is sequence identity measured by sequence alignment, while SeqID_aligned is sequence identity of the structurally aligned part.

**Table S16**. The list of protein chain pairs from the different crystal with <100% sequence identity, dataset set5. Reso1 and Reso2 are resolutions of the two structures, Len1 and Len2 are lengths of the two structures, SeqId is sequence identity measured by sequence alignment, while SeqID_aligned is sequence identity of the structurally aligned part. N-mut is the number of mutations in the N-terminal, mid-mut and C-mut are mutations in the middle and in the C-terminal respectively.
